# Exploitation of the Complement System by Oncogenic Kaposi's Sarcoma-Associated Herpesvirus for Cell Survival and Persistent Infection

**DOI:** 10.1371/journal.ppat.1004412

**Published:** 2014-09-25

**Authors:** Myung-Shin Lee, Tiffany Jones, Dae-Yong Song, Jae-Hyuk Jang, Jae U. Jung, Shou-Jiang Gao

**Affiliations:** 1 Department of Molecular Microbiology and Immunology, Keck School of Medicine, University of Southern California, Los Angeles, California, United States of America; 2 Department of Microbiology and Immunology, Eulji University School of Medicine, Daejeon, Republic of Korea; 3 Department of Anatomy and Neuroscience, Eulji University School of Medicine, Daejeon, Republic of Korea; Oregon Health and Science University, United States of America

## Abstract

During evolution, herpesviruses have developed numerous, and often very ingenious, strategies to counteract efficient host immunity. Specifically, Kaposi's sarcoma-associated herpesvirus (KSHV) eludes host immunity by undergoing a dormant stage, called latency wherein it expresses a minimal number of viral proteins to evade host immune activation. Here, we show that during latency, KSHV hijacks the complement pathway to promote cell survival. We detected strong deposition of complement membrane attack complex C5b-9 and the complement component C3 activated product C3b on Kaposi's sarcoma spindle tumor cells, and on human endothelial cells latently infected by KSHV, TIME-KSHV and TIVE-LTC, but not on their respective uninfected control cells, TIME and TIVE. We further showed that complement activation in latently KSHV-infected cells was mediated by the alternative complement pathway through down-regulation of cell surface complement regulatory proteins CD55 and CD59. Interestingly, complement activation caused minimal cell death but promoted the survival of latently KSHV-infected cells grown in medium depleted of growth factors. We found that complement activation increased STAT3 tyrosine phosphorylation (Y705) of KSHV-infected cells, which was required for the enhanced cell survival. Furthermore, overexpression of either CD55 or CD59 in latently KSHV-infected cells was sufficient to inhibit complement activation, prevent STAT3 Y705 phosphorylation and abolish the enhanced survival of cells cultured in growth factor-depleted condition. Together, these results demonstrate a novel mechanism by which an oncogenic virus subverts and exploits the host innate immune system to promote viral persistent infection.

## Author Summary

The complement system is an important part of the innate immune system. Pathogens have evolved diverse strategies to evade host immune responses including attack of the complement system. Kaposi's sarcoma-associated herpesvirus (KSHV) is associated with Kaposi's sarcoma (KS), primary effusion lymphoma and a subset of multicentric Castleman's disease. KSHV encodes a number of viral proteins to counter host immune responses during productive lytic replication. On the other hand, KSHV utilizes latency as a default replication program during which it expresses a minimal number of proteins to evade host immune detection. Thus, the complement system is expected to be silent during KSHV latency. In this study, we have found that the complement system is unexpectedly activated in latently KSHV-infected endothelial cells and in KS tumor cells wherein KSHV downregulates the expression of CD55 and CD59 complement regulatory proteins. More interestingly, most of latently KSHV-infected cells not only are resistant to complement-mediated cell killing, but also acquire survival advantage by inducing STAT3 tyrosine phosphorylation. These results demonstrate a novel mechanism by which an oncogenic virus exploits the host innate immune system to promote viral persistent infection.

## Introduction

Kaposi's sarcoma-associated herpesvirus (KSHV), also known as human herpesvirus 8 (HHV8), is a DNA tumor virus associated with the development of Kaposi's sarcoma (KS), primary effusion lymphoma (PEL), and a subset of multicentric Castleman's disease [1]–[3]. KS is a vascular tumor of proliferative endothelial cells displaying vast inflammation and uncontrolled angiogenesis while PEL and MCD are lymphoproliferative diseases of B-cells [4], [5].

The life cycle of KSHV consists of latency and lytic replication phases [6]. Following acute infection, KSHV establishes latency in the immunocompetent hosts. During latency, KSHV replicates in the episome form expressing only a limited number of viral latent proteins. As a result, KSHV latency is an effective strategy for evading host immune surveillance and maintaining persistent infection [6], [7]. In contrast, during lytic replication, KSHV replicates as a linear genome expressing cascades of viral proteins and producing infectious virions, which are exposed to host immune surveillance [6], [7]. In KSHV-related tumors, KSHV sustains a persistent infection with most tumor cells in latency and a small number of them undergoing lytic replication [5]. Therefore, KSHV latency is paramount to persistent infection in hosts with and without pathological manifestations.

KSHV maintains latency by evolving effective mechanisms for episome persistence, silencing expression of viral lytic genes, and promotion of survival and proliferation of infected cells [6]. A number of cellular signaling pathways including NF-κB, β-catenin, PI3K/AKT, c-Myc and ERK MAPK pathways are implicated in the growth and survival of latently KSHV-infected cells [8]–[13]. The STAT3 pathway, which can be activated by both KSHV-encoded IL-6 (vIL-6) and cellular IL-6, is essential for the survival of PEL cells [14], [15]. Although it is activated without involving vIL-6 and IL-6, the role of STAT3 pathway in the survival of latently KSHV-infected endothelial cells remains unclear [16].

The complement system consisting of over sixty components and activation fragments is a major arm of the innate immune system and plays important roles in stimulating inflammatory reactions, opsonization, complement-mediated cell killing, and induction of humoral and cell-mediated immune responses [17]–[20]. There are three distinct complement pathways including classical, lectin and alternative complement pathways. The classical complement pathway is activated by immune complexes involving the interaction of a minimum of two globular regions of C1q with the CH2 domains of an IgG duplex or a single IgM molecule [20], [21]. Thus, C1q is required for the activation of this pathway. The lectin pathway is initiated by binding of mannose-binding lectin to carbohydrate groups on the surface of bacterial cells [20], [21]. Unlike other complement pathways, the alternative complement pathway is spontaneously and constantly activated by the C3 complement protein, which itself is the recognition molecule [20], [21]. Tiny amounts of C3 are spontaneously hydrolyzed to C3(H_2_O) generating C3 convertase (C3bBb) with factor B and factor D [21]. Activation of the classical and lectin pathways, on the other hand, generates C4bC2a convertase, which is composed of C4b and C2a. Both C3bBb and C4bC2a convertases cleave C3 to C3a and C3b, also involving factor B and factor D [20], [21]. Addition of C3b to C3bBb and C4bC2a leads to the generation of C5 convertases C3bBbC3b and C4bC2aC3b, respectively, which cleave C5 to C5a and C5b [20], [21]. This initiates the terminal pathway, which leads to the formation of terminal complement complex, also known as MAC or C5b-9 complex consisting of C5b, C6, C7, C8 and C9 [20], [21].

Under normal conditions, complement regulatory proteins, which include soluble proteins and membrane-associated proteins, inhibit the activation of complement to protect cells from complement attack [20]–[22]. Soluble proteins include factor H and its related proteins and variants, and factor I while membrane-associated proteins include CD35, CD46, CD55, and CD59 [20]–[22]. In particular, factor H together with factor I bind to and cleave C3b in the alternative complement pathway, effectively preventing its further activation [20], [21], [23]. The membrane-associated protein CD55 primarily binds to C3b to prevent the activation of the complement pathway while CD59 binds to C5b-8 to prevent the formation of the C5b-9 complex [20], [21]. Blood-contacting cells such as red blood cells, white blood cells, and endothelial cells express high levels of complement regulatory proteins on their surfaces. If complement regulatory proteins are downregulated, the complement system can be activated [18], [24]–[26].

During complement activation, C3, C5 and their activation products in addition to the C5b-9 complex bind to cell surface receptors to trigger diverse biological effects including induction of inflammation, and cell growth and survival if the complexes are present at sublytic concentrations [18], [21], [27], [28]. Killing of target cells and pathogens upon activation is primarily mediated by the end product of the complement system, the C5b-9 complex, which forms transmembrane channels on the target cells [18], [19]. These channels disrupt the phospholipid bilayer of target cells, leading to cell lysis and death. Nucleated cells are more resistant to complement than erythrocytes or prokaryotes [20], [21], [29]. Killing of nucleated cells is a multi-hit process and requires formation of multiple channels on the cell membrane [20], [21], [29]. When formation of MAC on the cell surface is unable to lyse the target cells, MAC can trigger other biological effects [18], [29]–[33]. As a result, deregulation of the complement system has been linked to diverse pathological processes including oncogenic signal transduction associated with neoplastic progression, activation of cell cycle, enhanced angiogenesis, and resistance to apoptosis [18], [34], [35].

In this study, we have unexpectedly discovered that KSHV activates the complement system during latency. Interestingly, most of the latently KSHV-infected cells are resistant to complement-mediated cell killing. Furthermore, complement activation promotes cell survival of KSHV-infected cells cultured in medium without growth factors by activating the STAT3 pathway. These observations illustrate a strategy by which KSHV exploits the complement system for cell survival and persistent infection. Furthermore, we have observed complement activation in KS tumors indicating a role for the complement in promoting inflammation and angiogenesis during KSHV-induced tumorigenesis.

## Results

### The complement system is activated in KS tumors and in latently KSHV-infected endothelial cells

**Figure 1:**
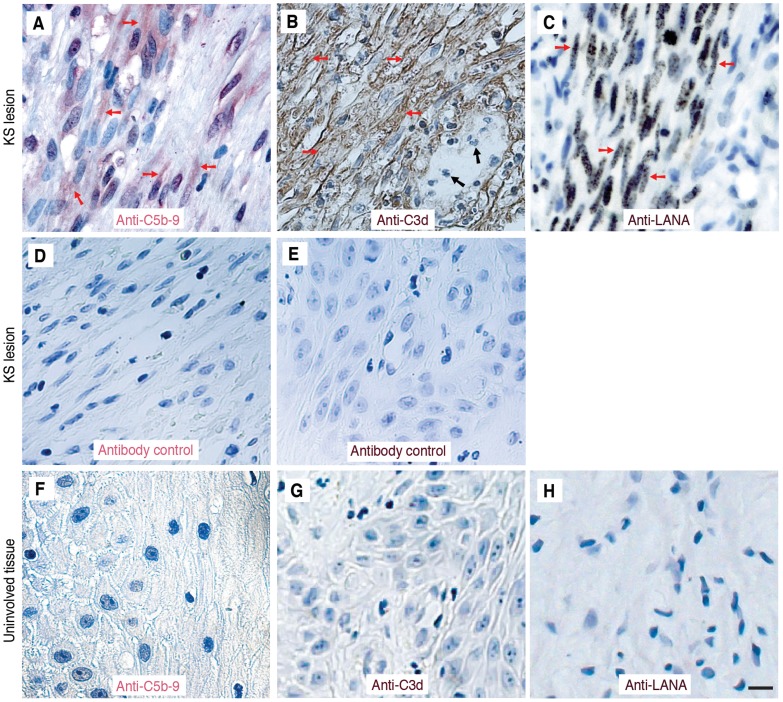
Detection of C5b-9 and C3d depositions on KS spindle tumor cells. (A–C) Representative illustration of immunohistochemical detection of C5b-9 deposition (A), C3d deposition (B) and KSHV latent protein LANA (C) in human KS tumors. Typical KS tumor spindle cells are indicated by red arrows. Black arrows show C3d-negative infiltrated immune cells (B). (D–E) Negative staining in KS tumors using matched non-immune sera for C5b-9 (D) and C3d (E) antibodies, respectively. (F–H) Negative control staining for C5b-9 deposition (F), C3d deposition (G) and LANA (H) in the uninvolved adjacent tissues using the respective antibodies. The scale bar is 10 µm.

During the process of investigating the mechanism of KSHV immune evasion, we unexpectedly found that KSHV activated the complement system during latency. In KS lesions, strong staining of C5b-9 was observed on the spindle tumor cells (Figure 1A). Consistent with C5b-9 staining, strong staining for C3d was also observed on the spindle tumor cells (Figure 1B), which were identified by positive staining for the major KSHV latent protein LANA (ORF73) (Figure 1C) [36]. A non-immune rabbit serum did not detect any positive signal in the KS lesions (Figure 1D and 1E). No C5b-9, C3d or LANA staining was observed in the healthy tissues adjacent to the KS lesions (Figure 1F, 1G and 1H). C3d is part of C3b, which is the central product of all three complement activation pathways while C5b-9 is the final product of all three complement activation pathways [18], [20], [21]. Detection of C3b and C5b-9 deposition on the tumor cells indicates activation of the complement system in KS tumors.

**Figure 2:**
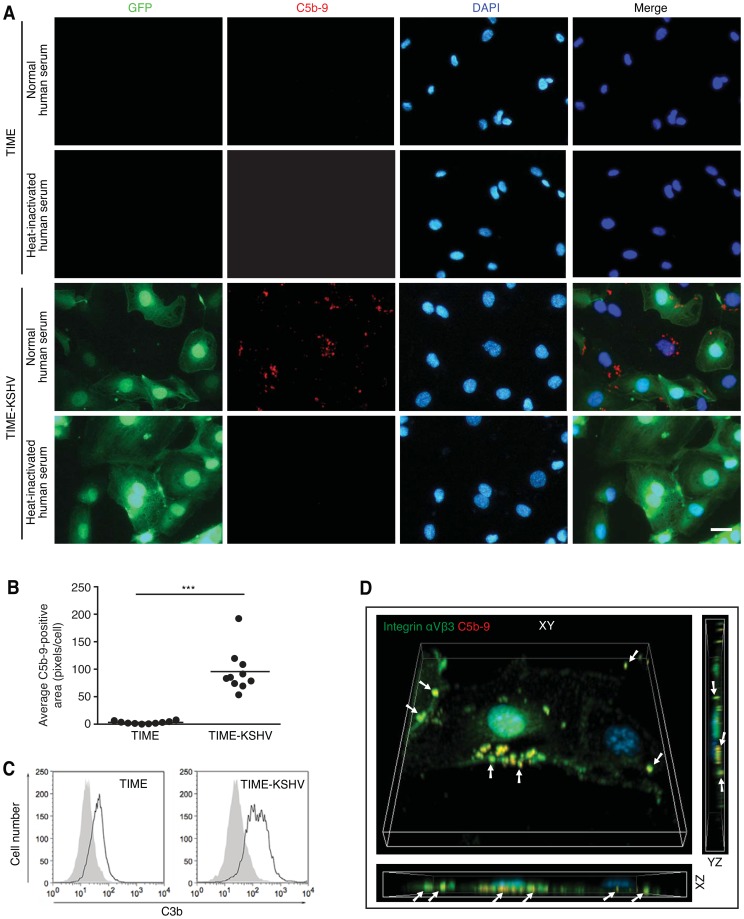
Detection and quantification of C5b-9 and C3b depositions on latently KSHV-infected endothelial cells. (A) Detection of C5b-9 deposition on TIME-KSHV but not TIME cells. Cells were incubated with 10% normal or heat-inactivated human serum for 30 min and stained for C5b-9 deposition by immunofluorescence staining. The scale bar is 50 µm. (B) Quantification of C5b-9 deposition on TIME and TIME KSHV cells. Ten images from each treatment shown in Figure S2 were analyzed for C5b-9-positive areas using the ImageJ software. The average C5b-9 staining area per cell was calculated by dividing the total pixels of C5b-9 staining in a microscopic field by the cell number. Each dot represents the analyzed value from one field. The average values of ten analyses were indicated as horizontal black bars. *** P<0.001. (C) Flow cytometry analysis of C3b staining on TIME and TIME-KSHV cells. White areas are C3b-positive cells. Grey areas are cells stained with an isotype-matched antibody control. (D) Cell surface localization of C5b-9 deposition on TIME-KSHV cells. TIME-KSHV cells were incubated with 10% normal human serum for 30 min, and stained for C5b-9 (red), integrin αVβ3 (captured in the far-red channel but pseudo-colored in green to facilitate visualization) to label plasma membrane and DAPI (blue) to localize the nucleus. Z-stack images were acquired with confocal laser-scanning microscopy. Three-dimensional software was used to generate z-projection images from at least 70 confocal images of 0.1 µm sections. The 3-D images (XY panels) were rotated on the x-axis (XZ panels) and y-axis (YZ panels) to visualize C5b-9 localization on the cell membrane. Arrows show representative areas of C5b-9 depositions on cell surfaces. Images of TIME cells incubated with normal human serum, and TIME and TIME-KSHV cells incubated with heat-inactivated serum are shown in Figure S3.

To determine if KSHV infection caused the activation of the complement system, we examined telomerase-immortalized microvascular endothelial cells (TIME) and TIME cells latently infected by a recombinant KSHV BAC36 (TIME-KSHV) [37]. While KSHV is inefficient in establishing persistent infection in TIME cells because of the rapid loss of viral episome [38], BAC36 contains a hygromycin-resistant cassette enabling the selection of stable latently KSHV-infected cultures. BAC36 also contains a green fluorescent protein (GFP) cassette facilitating the monitoring of KSHV infection. TIME-KSHV cells were latently infected by KSHV based on the expression of viral latent protein LANA and the lack of expression of lytic proteins K8.1 and ORF65 (Figure S1). Robust C5b-9 deposition was observed on TIME-KSHV cells while only weak or no C5b-9 staining was observed on TIME cells following incubation with medium containing normal human serum, which contained different components of the complement system (Figure 2A). As expected, we did not observe any C5b-9 deposition on TIME-KSHV and TIME cells when heat-inactivated human serum were used (Figure 2A).

We further quantified C5b-9 deposition on TIME and TIME-KSHV cells exposed to normal human serum. Ten random images from each cell type were quantified for C5b-9 deposition using the ImageJ's quantification tool (Figure S2) and the average pixels per cell of C5b-9-positive staining were presented (Figure 2B). While TIME cells had no or only few weak tiny C5b-9 particles, which probably reflected the background staining, all TIME-KSHV cells had C5b-9 depositions, many of which had strong large C5b-9-positive areas (Figure 2B). There was no correlation between C5b-9 deposition and GFP intensity of the cells. Flow cytometry analysis also detected weak C3b staining on the TIME cells and a much robust staining on the TIME-KSHV cells (Figure 2C).

To determine the cellular location of C5b-9 deposition, we stained integrin α5β3, which was primarily located in the cell plasma membrane. Z-stacks were acquired with a confocal laser-scanning microscope and used to generate XY 3-dimentional (3-D) projection overview images and the corresponding cross-sectional XZ and YZ images (Figure 2D and S3). C5b-9 deposition was primarily observed on the outer edges of cell plasma membranes (arrow). In some areas, C5b-9 localization on the cell surface was not obvious on the XY plane albeit their colocalization with α5β3. However, careful examination of the XZ and YZ planes clearly confirmed their cell surface localization.

KSHV lytic replication and the expression of viral lytic proteins can potentially trigger or amplify complement activation. C5b-9 and C3b depositions did not induce KSHV lytic replication as all KSHV-infected cells remained negative for viral early lytic protein ORF59 and late lytic protein ORF65 (Figure S4).

Similar results were also observed in long-term latently KSHV-infected telomerase-immortalized umbilical vein endothelial cells (TIVE-LTC), another KSHV latent infection model [39]. C5b-9 deposition was observed on TIVE-LTC but not on uninfected TIVE cells upon exposure to normal human serum (Figure S5). Together, these results suggest that KSHV might induce activation of the complement system during latency.

### Activation of the complement system in latently KSHV-infected endothelial cells is mediated by the alternative complement pathway

**Figure 3:**
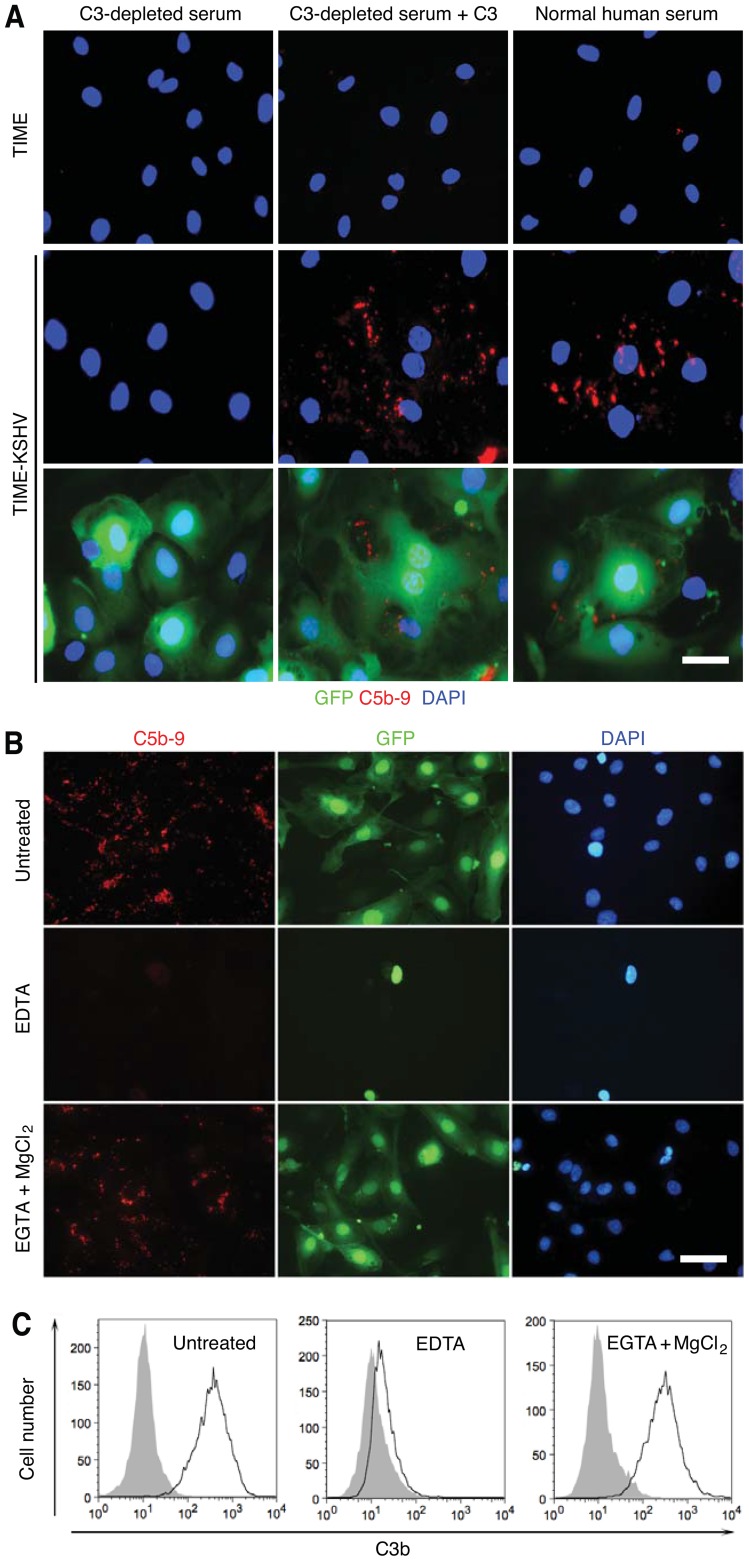
C5b-9 deposition on latently KSHV-infected endothelial cells is mediated by the alternative complement pathway. (A) C3 protein was essential for C5b-9 deposition on TIME-KSHV cells. TIME or TIME-KSHV cells were incubated for 30 min with C3-depleted human serum or C3-depleted human serum reconstituted with purified C3 protein. Normal human serum was used as a positive control. Immunofluorescence staining was performed to detect C5b-9 deposition. The scale bar is 50 µm. (B–C) Activation of complement in TIME-KSHV cells was sensitive to EDTA but resistant to EGTA and MgCl2. TIME-KSHV cells were incubated with normal human serum with or without the presence of 10 mM EGTA and 20 mM MgCl_2_ or 20 mM EDTA. C5b-9 deposition was detected by immunofluorescence staining (B) and C3b was detected by flow cytometry analysis (C), respectively. The scale bar is 100 µm.

C3 is essential for the activation of all three complement pathways [21]. To determine whether C5b-9 deposition on latently KSHV-infected cells was indeed dependent on activation of the complement system, we exposed TIME-KSHV cells to normal human serum depleted of the C3 component to inactivate all the complement pathways. Depletion of C3 component abolished C5b-9 deposition on TIME-KSHV cells (Figure 3A). However, addition of purified C3 proteins to the C3-depleted serum restored C5b-9 deposition. These results indicated that the observed C5b-9 deposition on latently KSHV-infected cells was specific and was indeed mediated by the complement system.

To identify the specific complement pathway activated by KSHV-infected cells, we exposed the cells to normal human serum in the presence of 10 mM EGTA and 20 mM MgCl_2_, which spared the alternative pathway but inactivated the other pathways, or 20 mM EDTA, which inactivated the alternative pathway [40], [41]. Addition of EGTA and MgCl_2_ to normal human serum did not affect the detection of C5b-9 and C3b on KSHV-infected cells (Figure 3B and 3C). However, addition of EDTA abolished the detection of C5b-9 and C3b. C1q is the initiation factor for the classical complement pathway while factor B is required for the initiation of the alternative complement pathway as well as amplification of all three complement pathways [20], [21]. As expected, depletion of C1q from the normal human serum had no effect on C5b-9 deposition on TIME-KSHV cells while depletion of factor B from the normal human serum abolished C5b-9 deposition (Figure S6). Together, these results indicated that alternative complement pathway was activated by latently KSHV-infected cells.

### Complement regulatory proteins are downregulated in latently KSHV-infected endothelial cells in culture and in KS tumor cells

**Figure 4:**
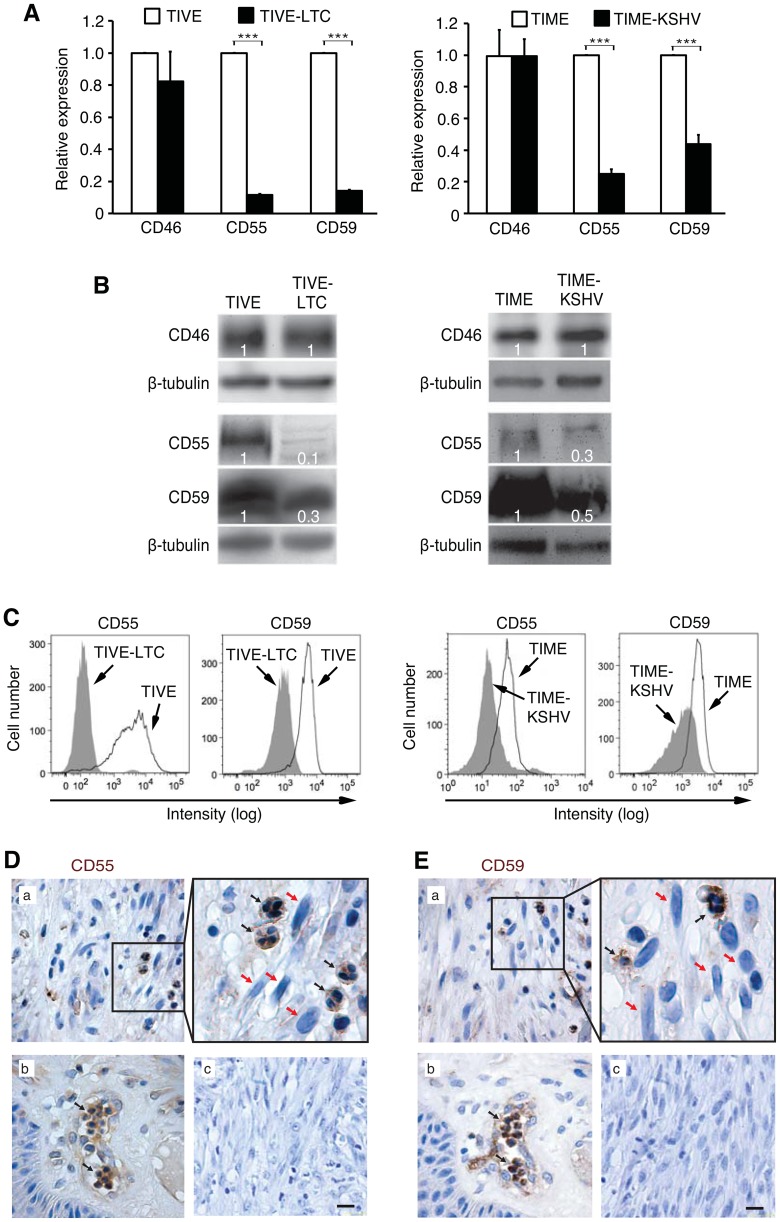
Complement regulatory proteins CD55 and CD59 but not CD46 are downregulated on latently KSHV-infected endothelial cells and on KS tumor cells. (A) Relative expression levels of CD46, CD55 and CD59 mRNAs in uninfected and KSHV-infected endothelial cells. The level of mRNA expression was measured by RT-qPCR. GAPDH gene was used as a calibration control. The expression levels of uninfected cells were set as “1”. Results shown as means ± SD are representative from three independent experiments. * P<0.05, ** P<0.01 and *** P<0.001 by Student's *t*-test. (B) Total protein levels of CD46, CD55 and CD59 were examined by Western-blotting using antibodies to CD46, CD55 and CD59, respectively. (C) Surface protein levels of CD55 and CD59 on uninfected and KSHV-infected endothelial cells were analyzed by flow cytometry. (D–E) Representative illustration of detection of CD55 (D) and CD59 (E) in KS tumors. (a) Paraffin-embedded KS tumor sections with typical spindle cells were stained with antibodies to C55 and CD59. Representative areas are shown in the top right panel. Typical KS tumor spindle cells are indicated by red arrows while immune cells are indicated by black arrows. (b) Uninvolved adjacent tissues with endothelial cells and mononuclear cells were stained for CD55 and CD59 as positive controls. Representative mononuclear cells were indicated with black arrows. (c) Negative control staining with the isotype-matched control antibodies in the same KS tumors as (a). The scale bar is 10 µm.

Because we detected most of the C5b-9 deposition on the spindle tumor cells rather than across the entire tumor section in the immunohistochemical staining (Figure 1), we focused on the cell surface-expressing complement regulatory proteins. Reverse transcription real-time PCR (RT-qPCR) detected strong expression of CD55 and CD59 in TIME or TIVE cells (Figure 4A), which were consistent with the absence of complement activation on these cells upon exposure to normal human serum (Figure 2 and S5). KSHV infection significantly downregulated the expression of CD55 and CD59 in TIME-KSHV and TIVE-LTC cells (Figure 4A). In contrast, we detected no difference of expression for CD46 between uninfected and KSHV-infected cells (Figure 4A), and no expression of CD35 in any of the cells tested, suggesting that both CD46 and CD35 were not involved in the control of complement activation in these cells. Consistent with the mRNA results, total CD55 and CD59 proteins detected by Western-blotting and their cell surface expression levels detected by flow cytometry were expressed in much lower levels in KSHV-infected endothelial cells than in the uninfected cells (Figure 4B–4C). As expected, there was no difference of CD46 protein expression between uninfected and KSHV-infected cells (Figure 4B). These results indicated that KSHV infection might activate the alternative complement pathway by downregulating the expression of CD55 and CD59 proteins. To further determine the biological relevance of these observations, we examined the expression CD55 and CD59 proteins in KS tumors (Figure 4D–4E). In agreement with the cell culture results, we observed weak or close to no expression of CD55 and CD59 on the typical spindle tumor cells in KS tumors (red arrows in Figure 4D–4E: a). However, CD55 and CD59 were highly expressed in the infiltrated immune cells surrounding the spindle cells (black arrows in Figure 4D–4E: a) or in normal blood vessels adjacent to the tumors (black arrows in Figure 4D–4E: b). No staining was observed in the KS tumors stained with isotype-matched control antibodies (Figure 4D–4E: c).

### Latently KSHV-infected endothelial cells are resistant to complement-mediated cytolysis

**Figure 5:**
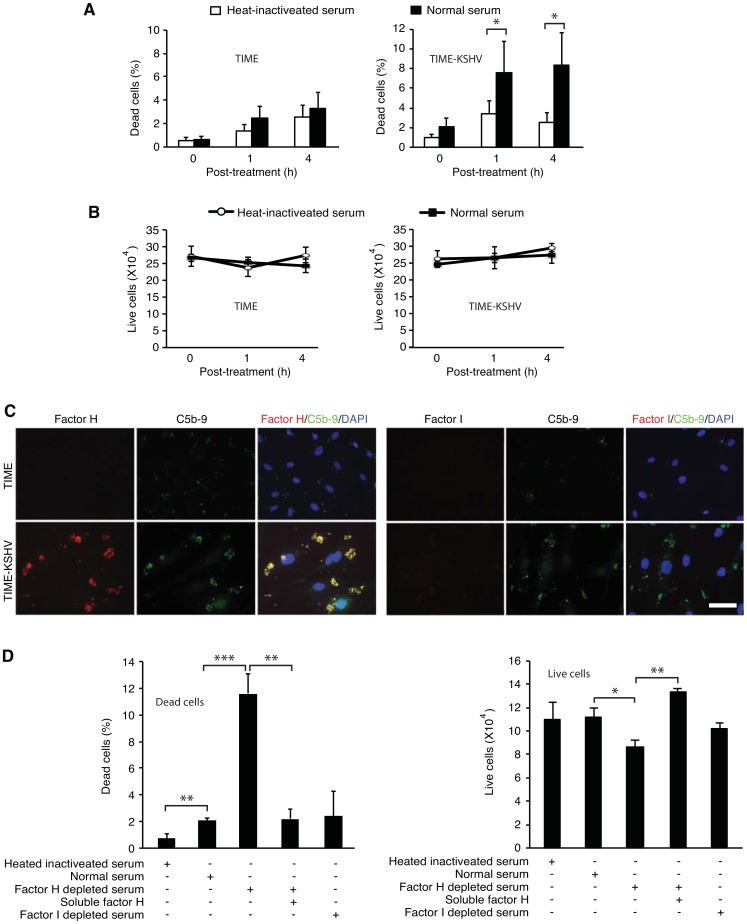
Factor H is required for resistance to complement-mediated cytolysis of latently KSHV-infected endothelial cells. (A–B) Most of latently KSHV-infected endothelial cells are resistant to complement-mediated cytolysis. TIME or TIME-KSHV cells were incubated with 10% normal human serum or heat-inactivated human serum for 1 h and 4 h, and the number of dead cells (A) and live cells (B) were determined. (C) Deposition of factor H but not factor I on TIME-KSHV cells following complement activation. TIME or TIME KSHV cells were incubated for 30 min with normal human serum, and stained for C5b-9 (green) and factor H (red in left panel) or factor I (red in right panel). Note the colocalization of factor H with C5b-9. The scale bar is 80 µm. (D) Factor H was required for resistance to complement-mediated cytolysis. TIME-KSHV cells were incubated for 1 h with heat-inactivated human serum, normal human serum, factor H-depleted human serum, factor H-depleted human serum reconstituted with purified factor H protein or factor I-depleted human serum, and the number of dead cells (left panel) and live cells (right panel) were determined. All the experiments were performed in 6 well plates with 6 repeats. Results are means ± SD from two to three independent experiments. * P<0.05 and ** P<0.01 by Student's *t*-test.

C5b-9 induces cell lysis by assembling a pre-forming complex on the cell membrane [18], [34]. Because we observed C5b-9 deposition on latently KSHV-infected endothelial cells (Figure 2), we further examined the fate of these cells. Both TIME and TIME-KSHV cells cultured in heat-inactivated human serum had low basal levels of dead cells in the range of 1–4% (Figure 5A). In agreement with the absence of C5b-9 deposition (Figure 2), there was no significant increase of dead cells in TIME cells exposed to normal human serum (Figure 5A). In contrast, the number of dead cells in TIME-KSHV cells exposed to normal human serum for 1 h was significantly higher than those exposed to heat-inactivated human serum under the same condition (7.5% *vs* 3.4%, P<0.05) (Figure 5A). The number of dead cells in TIME-KSHV cells following exposure to normal human serum might vary but was usually in the range of 5–12% depending on the batch of the serum. Extended exposure of the cells for up to 8 h or addition of new normal human serum to the medium to avoid possible complement exhaustion or inactivation in the medium did not further increase the number of dead cells. There was no detectable change of total live cells in both TIME and TIME-KSHV cells cultured in heat-inactivated and normal human serum (Figure 5B). These results indicated that most latently KSHV-infected cells were resistant to complement-mediated cytolysis.

Because KSHV lytic proteins ORF59 and ORF65 remained undetectable following exposure to the complement (Figure S4), we concluded that viral lytic replication did not influence the sensitivity of KSHV-infected cells to complement-mediated cytolysis. KSHV ORF4 encodes a complement inhibitory protein named KCP or kaposica that inhibits complement activation [42], [43]. ORF4 is a viral lytic protein. We did not detect any expression of ORF4 in KSHV-infected cells before and after complement exposure (Figure S7), indicating that this viral protein did not mediate the resistance of TIME-KSHV cells to the complement. We also did not detect any changes in CD55 and CD59 expression in KSHV-infected cells following complement exposure by either immunofluorescence staining or Western-blotting (Figure S8), indicating that these complement regulatory proteins did not mediate the resistance of KSHV-infected cells to the complement-mediated cytolysis. To further determine if the sensitivity of a small fraction of KSHV-infected cells to complement-mediated cytolysis was due to a larger amount of C5b-9 deposition on these cells, we quantified C5b-9 deposition on the individual live and dead cells following their exposure to normal human serum. Examination of 20 randomly selected live or dead cells revealed wide ranges of C5b-9 deposition on the individual cells in both live and dead cells albeit a few strong staining cells were observed on the dead cells (Figure S9 and S10). Thus, we concluded that the sensitivity of a small fraction of KSHV-infected cells to complement-mediated cytolysis was not due to a larger amount of C5b-9 deposition on these cells.

The generation of C3b and C3d as a result of C3 activation leads to the recruitment of soluble factor H and factor I on the cell surface [44]. In particular, C3d binds to factor H forming multimeric factor H-C3d complexes [45]. Indeed, strong factor H staining was detected on KSHV-infected cells but not on uninfected cells following exposure to normal human serum (Figure 5C). Interestingly, the majority of factor H signals were colocalized with those of C5b-9. Colocalization of factor H with C5b-9 was reported before in *de novo* membranous glomerulonephritis occurring in patients with renal transplant [46]. Unexpectedly, no factor I signal was detected on KSHV-infected or -uninfected cells following exposure to normal human serum (Figure 5C). Recruitment of factor H protects cells from further complement attack, which is amplified by factor I [20], [21]. Thus, we examined the roles factors H and I in protecting KSHV-infected cells from complement-mediated cytolysis. Depletion of factor H significantly increased the number of dead cells and reduced the number of live cells while addition of purified factor H to the depleted serum prevented the increase of dead cells (Figure 5D). In contrast, no obvious change in the number of live or dead cells was observed following the depletion of factor I. These results indicated that binding of factor H but not factor I conferred KSHV-infected cells resistance to complement-mediated cytolysis.

### Complement activation enhances the survival of latently KSHV-infected endothelial cells cultured in growth factor-depleted medium

**Figure 6:**
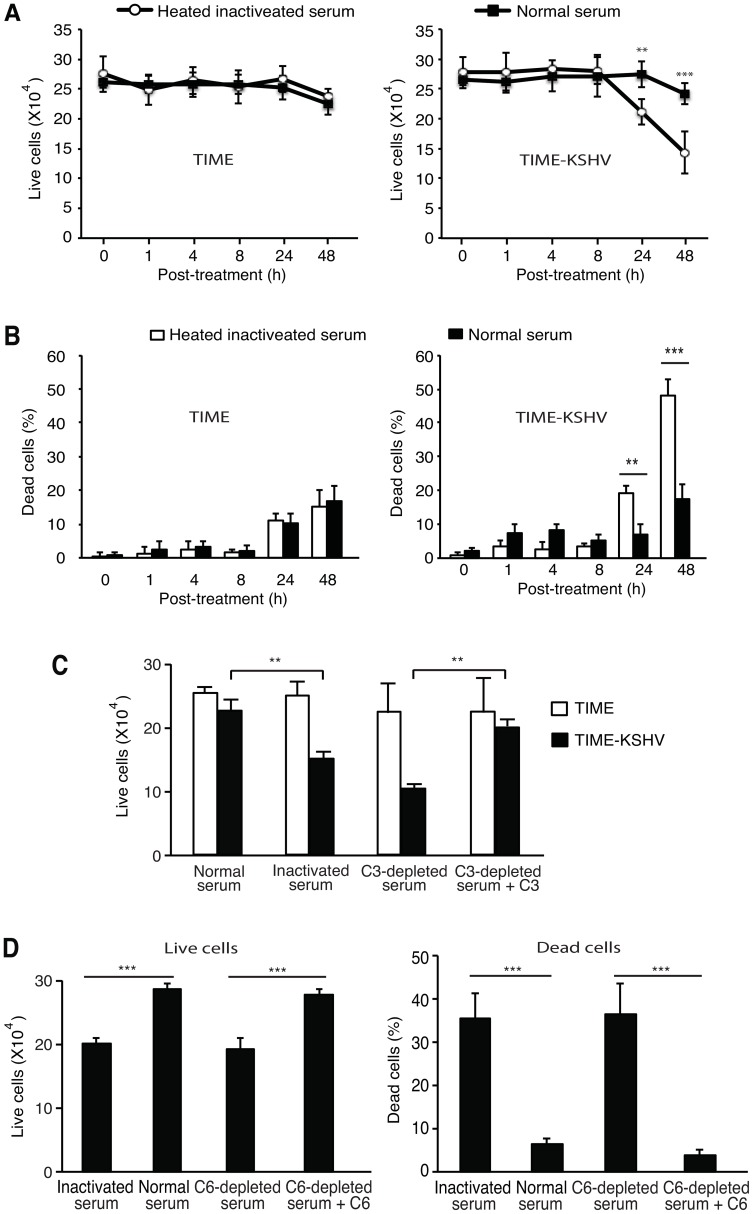
Complement activation promotes cell survival of latently KSHV-infected cells in growth factor-depleted medium. (A–B) Complement activation promotes cell survival of latently KSHV-infected cells. Cells were cultured in endothelial cell medium depleted of growth factors and 10% of heat-inactivated or normal human serum, and live cells (A) and dead cells (B) were determined at the indicated times. (C) C3 was required for the enhanced cell survival of TIME-KSHV cells cultured in growth factor-depleted medium. TIME and TIME-KSHV cells were cultured for 48 h in 10% C3-depleted human serum or C3-depleted human serum reconstituted with purified C3 protein in endothelial cell medium without growth factors, and the numbers of live cells were determined. Cells cultured in heat-inactivated and normal human sera were used as controls. (D) Terminal complement complex was required for the enhanced survival of TIME-KSHV cells cultured in growth factor-depleted medium. TIME-KSHV cells were cultured for 48 h in 10% C6-depleted human serum or C6-depleted human serum reconstituted with purified C6 protein in endothelial cell medium depleted of growth factors, and the numbers of live cells (left panel) and dead cells (right panel) were determined. Cells cultured in heat-inactivated and normal human sera were used as controls. Results are means ± SD from three independent experiments with three repeats. * P<0.05, ** P<0.01 and *** P<0.001 by Student's *t*-test.

Sublytic C5b-9/MAC has been associated with enhanced cell survival [19], [21]. Because most of latently KSHV-infected endothelial cells were resistant to complement-mediated cell killing (Figure 5A–5B), we determined the effect of the activated complement on cell survival. Cells were cultured in medium depleted of growth factors and heat-inactivated or normal human serum. The total number of live cells in TIME cells cultured in both heat-inactivated and normal human sera remained unchanged for up to 24 h, and then decreased by 8–10% at 48 h (Figure 6A). Consistent with these results, the number of dead cells in TIME cells cultured in heat-inactivated and normal human sera increased from the basal 1% and 2% to 15% and 17% at 48 h, respectively (Figure 6B). These results indicated that most of TIME cells could survive for up to 48 h without growth factors. Furthermore, there was no obvious difference between TIME cells cultured in heat-inactivated and normal human sera. In contrast, TIME-KSHV cells cultured in heat-inactivated human serum had a significant decrease in the number of live cells by 25% and 50% at 24 h and 48 h, respectively (Figure 6A). The number of dead cells also increased from the basal 2% to 19% by 24 h and to 48% by 48 h, respectively (Figure 6B). These results indicated that TIME-KSHV cells were more sensitive to growth factor depletion than TIME cells when both types of cells were cultured in heat-inactivated human serum. However, the sensitivity of TIME-KSHV cells cultured in heat-inactivated human serum to growth factor depletion could be rescued by normal human serum. Specifically, TIME-KSHV cells cultured in normal human serum had no detectable change in the number live cells by 24 h and had only a slight decrease by 11% at 48 h (Figure 6A). Following an initial increase from the basal 2% to 6–8% as shown in Figure 5A, the number of dead cells in TIME-KSHV cells cultured in normal human serum remained at 7% at 24 h and was increased to 18% at 48 h, which was significantly lower than the 48% in the same cells cultured in heat-inactivated human serum (P<0.001 in Figure 6B). These results suggested that a large portion of TIME-KSHV cells failed to survive without growth factors but normal human serum could protect them from cell death and extend their survival.

To exclude possible confounding effect of growth factors in the normal human serum, we cultured the cells in medium with C3-depleted human serum and the same depleted serum reconstituted with purified C3 protein. Similar numbers of live cells were observed for TIME cells cultured in medium with and without C3 protein at 48 h (Figure 6C). However, the number of live cells in TIME-KSHV cells cultured in C3-depleted serum was significantly lower than those cultured in C3-reconstituted serum (P<0.01 in Figure 6C). These results confirmed that activation of the complement pathway promotes cell survival of latently KSHV-infected endothelial cells cultured in growth factor-depleted medium.

To identify the complement protein(s) that regulates the survival of latently KSHV-infected endothelial cells, we cultured the cells in medium with C6-depleted human serum. Similar to depletion of C3 protein, depletion of C6 protein decreased the number of live cells but increased the number of dead cells (Figure 6D). Addition of purified C6 protein to the depleted serum, which reconstituted the complement system, prevented cell death (Figure 6D). These results indicated that the late complement components, C5b to C9, and their complexes were important for the survival of latently KSHV-infected endothelial cells following growth factor depletion.

### Activation of the alternative complement pathway promotes the survival of latently KSHV-infected endothelial cells by inducing STAT3 phosphorylation

**Figure 7:**
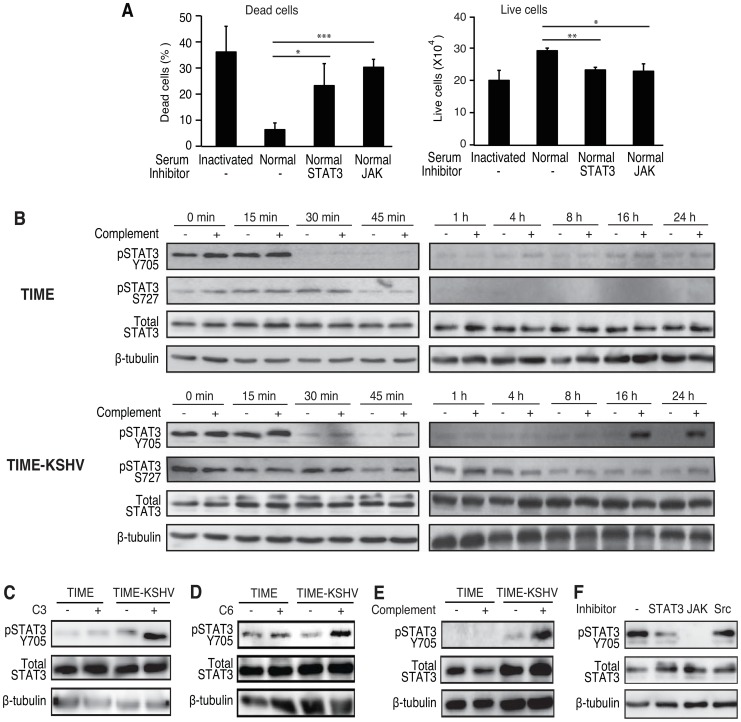
Activation of complement pathway induces STAT3 phosphorylation to enhance cell survival of latently KSHV-infected endothelial cells. (A) The enhanced cell survival of KSHV-infected endothelial cells by complement was mediated by the STAT3 pathway. TIME-KSHV cells were cultured for 48 h in normal human serum in growth factor-depleted medium with and without JAK or STAT3 inhibitor, and the numbers of dead cells (left panel) and live cells (right panel) were determined. Cells cultured in heat-inactivated human serum were used as controls. Results are means ± SD from three independent experiments with three repeats. * P<0.05, ** P<0.01 and *** P<0.001 by Student's *t*-test. (B) Complement activation induced STAT3 tyrosine phosphorylation in TIME-KSHV cells. STAT3 tyrosine (Y705) and serine (S727) phosphorylation in TIME and TIME-KSHV cells switched from full endothelial cell medium with growth factors and 10% heat-inactivated human serum to endothelial cell medium depleted of growth factors with 10% heat-inactivated or normal human serum for the specified lengths of time. (C) Complement activation was required for the enhanced STAT3 tyrosine phosphorylation of TIME-KSHV cells. STAT3 tyrosine phosphorylation was examined in cells cultured for 24 h in 10% C3-depleted human serum or C3-depleted human serum reconstituted with purified C3 protein in endothelial cell medium depleted of growth factors. (D) Formation of C5b-9 complexes was required for the enhanced STAT3 tyrosine phosphorylation. STAT3 tyrosine phosphorylation was examined in cells cultured for 24 h in 10% C6-depleted human serum or C6-depleted human serum reconstituted with purified C6 protein in endothelial cell medium deprived of growth factors. (E) STAT3 tyrosine phosphorylation was unchanged without the continuous presence of normal human serum and growth factors. STAT3 tyrosine phosphorylation was examined in cells cultured in 10% heat-inactivated or normal human serum for 1 h, and then in endothelial cell medium without any serum and growth factors for additional 23 h. (F) JAK but not Src activation mediated STAT3 tyrosine phosphorylation. STAT3 tyrosine phosphorylations was examined in TIME-KSHV cells cultured in 10% normal human serum in endothelial cell medium depleted of growth factors for 24 h with or without the presence of STAT3, JAK or Src inhibitor.

Binding of the C5b-9 complexes to target cells can trigger acute activation of diverse signaling pathways including ERK1, JNK1, p38, PI3K/AKT, and JAK/STAT3 pathways [21]. To determine the pathway that mediated the enhanced survival of latently KSHV-infected endothelial cells grown in medium without growth factors, we examined the effects of normal human serum in the presence of specific inhibitors of different pathways activated by the C5b-9 complexes. Among all the inhibitors examined, only STAT3 and JAK inhibitors antagonized the enhanced survival effect of complement activation (Figure 7A and S11, S12). MEK and PLC inhibitors caused cell morphological changes, which were probably due to their toxicity. However, they did not increase any cell death within the experimental timeframe (Figure S12). In contrast, STAT3 and JAK inhibitors increased the number of dead cells in TIME-KSHV cells cultured in normal human serum to the levels similar to those cultured in heat-inactivated serum (Figure 7A and S11). Consistent with these results, treatment with STAT3 and JAK inhibitors reduced the number of live cells in TIME-KSHV cells cultured in normal human serum to the levels similar to those cultured in heat-inactivated serum (Figure 7A). Importantly, there was no change in C5b-9 deposition following treatment with STAT3 inhibitor (Figure S13). These results suggested that the STAT3 pathway likely mediated the complement enhanced cell survival of TIME-KSHV cells grown in medium without growth factors.

We further examined the effect of complement activation on the STAT3 pathway. In full endothelial cell medium containing growth factors, we detected strong STAT3 Y705 phosphorylation in both TIME and TIME-KSHV cells (0 min in Figure 7B). TIME-KSHV cells also had strong STAT3 S727 phosphorylation but much weaker signal was observed in TIME cells (0 min in Figure 7B). These results are not surprising because a number of growth factors such as EGF and PDGF in the endothelial cell medium are known to activate STAT3 [47]. When we exchanged the medium for endothelial cell basal media without growth factors containing 10% heat-inactivated or normal human serum, Y705 phosphorylation decreased rapidly within 30 min in both TIME and TIME-KSHV cells (Figure 7B). Weak STAT3 Y705 phosphorylation persisted for up to 24 h in TIME cells cultured in either heat-inactivated or normal human serum. While STAT3 Y705 phosphorylation remained weak in TIME-KSHV cells cultured in heat-inactivated and normal human serum in the first 8 h, a significant increase of STAT3 Y705 phosphorylation was observed in these cells cultured in normal human serum but not those cultured in heat-inactivated serum by 16 h and 24 h (Figure 7B). STAT3 S727 phosphorylation also decreased in both TIME and TIME-KSHV cells by 45 min after the full endothelial cell medium was exchanged to basal medium without growth factors (Figure 7B). The S727 phosphorylation signal in TIME cells grown in heat-inactivated or normal human serum was almost not detectable by 1 h following the depletion of growth factors. Under the same condition, we continued to detect S727 phosphorylation in TIME-KSHV cells grown in heat-inactivated or normal human serum (Figure 7B). These results are consistent with the observation of kaposin B activation of S727 but not Y705 phosphorylation in primary human endothelial cells latently infected by KSHV [48].

To confirm that the increased STAT3 Y705 phosphorylation in TIME-KSHV cells following prolonged culture in normal human serum without growth factors was due to activation of the complement system, we examined the cells cultured for 24 h in medium with C3-depleted serum without growth factors. Depletion of C3 protein abolished the increase of STAT3 Y705 phosphorylation of TIME-KSHV cells while reconstitution of the C3 protein in the depleted serum effectively restored Y705 phosphorylation (Figure 7C), indicating that STAT3 Y705 phosphorylation was indeed mediated by complement activation. Similarly, depletion of C6 protein abolished the increase of STAT3 Y705 phosphorylation in TIME-KSHV cells while reconstitution of the C6 protein in the depleted serum effectively restored Y705 phosphorylation (Figure 7D), indicating that STAT3 Y705 phosphorylation was mediated by the C5b-9 complex. Moreover, when cells were exposed to normal human serum for 1 h and subsequently cultured in endothelial basal media without growth factors and without serum for 24 h, we could still detected the increased STAT3 Y705 phosphorylation in TIME-KSHV cells (Figure 7E), indicating that the effect of complement activation on STAT3 tyrosine phosphorylation was sustained for at least 24 h even without the persistent presence of normal human serum and growth factors.

STAT3 tyrosine phosphorylation is mediated by Janus kinases (JAKs) in response to activation of cytokine receptors or by Src in response to growth factor receptors [47]. JAK inhibitor but not Src inhibitor blocked complement activation of STAT3 Y705 phosphorylation (Figure 7F), suggesting that JAKs mediated the observed STAT3 tyrosine phosphorylation, most likely through cytokines that were induced upon complement activation. Nevertheless, we cannot exclude the possible direct induction of STAT3 Y705 phosphorylation by the C5b-9 complexes.

### Overexpression of either CD55 or CD59 in latently KSHV-infected endothelial cells is sufficient to inhibit complement activation, prevent STAT3 Y705 phosphorylation and abolish the enhanced cell survival

**Figure 8:**
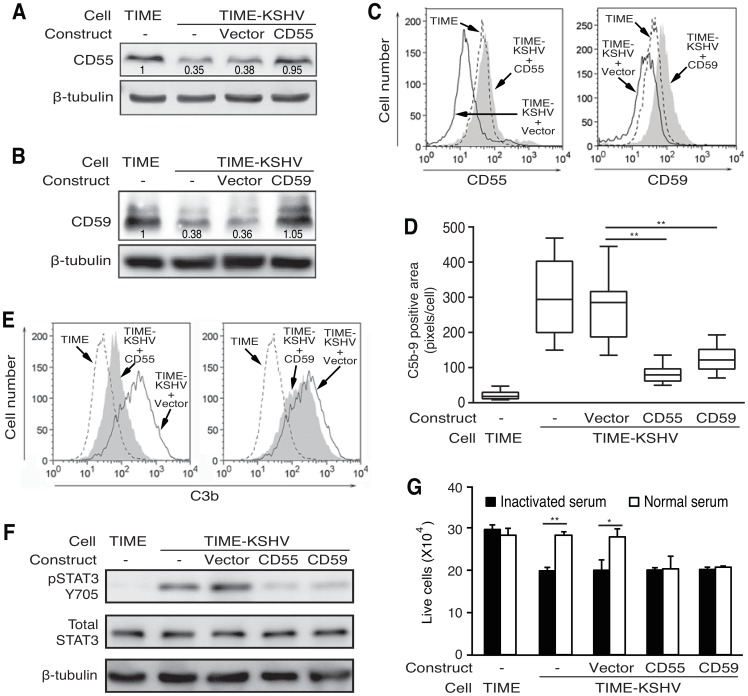
Overexpression of CD55 and CD59 in latently KSHV-infected endothelial cells abolishes complement activation, induction of STAT3 phosphorylation, and enhancement of cell survival. (A–B) Western-blot examination of CD55 (A) and CD59 (B) protein expression in TIME-KSHV cells with stable overexpression of CD55 and CD59. TIME cells were used as controls for the relative expression levels of CD55 and CD59. (C) Flow cytometry analysis of CD55 (left panel) and CD59 (right panel) cell surface expression on TIME-KSHV cells with stable overexpression of CD55 and CD59. TIME cells were used as controls for the relative expression levels of CD55 and CD59. (D) Quantification of C5b-9 deposition on TIME-KSHV cells with overexpression of CD55, CD59 or vector control. Box and whisker plots showing the mean (middle line) and 25–75^th^ percentiles (lower and top box lines) of the average C5b-9 deposited areas on TIME cells (n = 10), TIME-KSHV cells (n = 10), and TIME-KSHV cells with overexpression of CD55 (n = 10), CD59 (n = 10) or vector control (n = 10) following exposure to 10% normal human serum for 1 h. Top and lower lines indicate the maximal and minimal values. Ten images from each cell type shown in Figure S14 were analyzed for C5b-9-positive areas using the ImageJ software. The average C5b-9 staining area per cell was calculated by dividing the total pixels of C5b-9 staining in a microscopic field by the cell number. (E) Flow cytometry analysis of C3b deposition on TIME-KSHV cells with overexpression of CD55 (left panel) or CD59 (right panel). TIME cells and TIME-KSHV cells transduced with the vector were used as controls. (F) Overexpression of CD55 or CD59 abolished complement activation of STAT3 tyrosine phosphorylation in TIME-KSHV cells. STAT3 tyrosine phosphorylation was examined in cells cultured in 10% normal human serum for 24 h. (G) Overexpression of CD55 or CD59 abolished the enhanced cell survival in TIME-KSHV cells cultured in medium depleted of growth factors. Cells were cultured for 48 h in heat-inactivated or normal human serum in endothelial cell medium depleted of growth factors in the presence of the indicated serum, and live cells were determined. Results are means ± SD from three independent experiments with three repeats. * P<0.05 and ** P<0.01 by Student's *t*-test.

Our results so far suggested that downregulation of CD55 and CD59 expression might mediate complement activation in latently KSHV-infected endothelial cells (Figure 4). We therefore generated TIME-KSHV cells with stable overexpression of CD55 or CD59 protein. The levels of CD55 and CD59 proteins in these stable cells were similar to those in TIME cells based on the results of Western-blotting and flow cytometry analysis (Figure 8A–8C). CD55 primarily inhibits C3 activation and amplification of the activated complement system while CD59 inhibits C5b-9 formation [20], [21], [29]. As expected, overexpression of either CD55 or CD59 protein was sufficient to significantly decrease C5b-9 deposition on TIME-KSHV cells exposed to normal human serum (Figure 8D and S14). Overexpression of CD55 also significantly decreased C3b deposition on TIME-KSHV cells; however, overexpression of CD59, as expected, had minimal effect on C3b deposition on these cells (Figure 8E). Importantly, overexpression of either CD55 or CD59 protein was sufficient to reduce STAT3 Y705 phosphorylation in TIME-KSHV cells cultured for 24 h in growth factor-depleted medium containing normal human serum (Figure 8F). Consistent with these results, TIME-KSHV cells with overexpression of either CD55 or CD59 protein cultured in growth factor-depleted medium containing normal human serum no longer survived better than those cultured in heat-inactivated human serum (Figure 8G). Together, these results indicated that downregulation of CD55 and CD59 proteins mediated complement activation in latently KSHV-infected endothelial cells, which resulted in the activation of STAT3 and prevented cell death induced by growth factor depletion.

## Discussion

Viruses have evolved diverse mechanisms to evade the host immunity including the complement system [49]. During lytic replication, KSHV produces a large number of viral lytic proteins and virions, which are exposed to host immune surveillance. KSHV encodes several proteins to inhibit host immune responses and ensure successful infection and replication [7]. In particular, KSHV encodes a functional homologue of complement regulatory protein KCP [42], [43]. KSHV KCP has structure and function similar to CD55, also known as decay accelerating factor (DAF), which inhibits activation of the complement system. KCP is a viral lytic protein, which could protect KSHV virions if it is incorporated, and KSHV-infected cells when it is highly expressed, from complement attack during viral acute infection and lytic replication.

Although it encodes an arsenal of immune evasion molecules [7], KSHV establishes latency with minimal expression of viral proteins in immunocompetent host following acute infection. In KS tumors, most tumor cells are also latently infected by KSHV [5]. Thus, latency is the default program by which KSHV evades the host immunity [7].

In this study, we have unexpectedly found that KSHV activates the complement system during latency. In KS tumors, we have detected C5b-9 and C3d depositions on the LANA-positive spindle tumor cells. In culture, the complement system is activated in latently KSHV-infected endothelial cells upon exposure to normal human serum. Interestingly, most of KSHV-infected endothelial cells are resistant to complement-mediated cytolysis. Thus, there are specific mechanisms for both activating and evading the complement system by KSHV in the latently infected cells. Consistent with these cell culture results, KS spindle tumor cells are healthy, and are spared from complement attack in spite of C5b-9 deposition.

Among the three complement pathways, activation of the classical pathway is initiated by the formation of the antigen-antibody complex [18], [20], [21]. Binding of antibodies to viral proteins on the surface of virions or cells triggers the activation of the classical complement pathway. In latently KSHV-infected cells, only a few viral latent proteins are expressed, none of which is present on the surface of the infected cells [5]. Although the breakdown of cell membranes and loss of membrane complement regulators in apoptotic cells can activate the classical pathway by recruiting C1q and factor H, this would only initiate the complement cascade and generate C3b but block C3 and C5 convertases, and hence the formation of C5b-9 complexes [20]. We have detected few apoptotic cells (<3%) in latently KSHV-infected cells before their exposure to the normal human serum. Furthermore, we have shown that C1q is not required for the complement activation and there is strong C5b-9 deposition following complement activation in latently KSHV-infected cells. These results indicate that the classical complement pathway is unlikely activated during KSHV latent infection. The mannose-binding lectin pathway is activated by the terminal mannose groups present on the microbes, which are absent on latently KSHV-infected cells. Therefore, similar to the classical pathway, the lectin pathway is unlikely activated during KSHV latent infection. Distinct from other pathways, activation of the alternative complement pathway is more complex, which is often involved with more than one factor. Although bacteria, fungi and viruses are the primary initiators, tumor cells can also activate the alternative pathway though the precise mechanism remains unclear [50], [51]. We have shown that complement activation is not affected by the presence of 10 mM EGTA and 20 mM MgCl_2_ but abolished by the presence of 20 mM EDTA. Together, these results have shown that latently KSHV-infected cells specifically activate the alternative complement pathway.

It is possible that classical complement pathway can be activated in KS tumors if there is active viral lytic replication since most of KS patients have antibodies to KSHV lytic proteins [52], [53]. However, only a small percentage of the KSHV-infected cells are lytic cells in KS tumors, and in late stage of KS tumors, there is virtually no lytic cell [6]. Our results have shown that most of the spindle tumor cells are LANA-positive with less than 1% of ORF65-positive cells. Furthermore, there is no difference in C5b-9 or C3d staining in KS lesions with and without lytic cells suggesting that the small number of the lytic cells would unlikely be involved in the amplification of the complement cascade.

Complement activation is a complex process involving with more than sixty components and activation fragments [17]–[20]. Because complement regulatory proteins are important factors controlling the activation of the complement system, downregulation of these proteins often results in the activation of the complement system [20], [21], [24], [54]. Among them, factor H plays a critical role in regulating the activation of alternative complement pathway as it has been shown in the association of an increased risk of developing both early and late age-related macular degeneration (AMD) with a single nucleotide polymorphism in factor H [55]–[58]. However, down-regulation of other complement regulatory proteins such as CD55 and CD59 has also been shown to be involved in complement activation. In fact, downregulation or lack of CD55 and CD59 expression is associated with clinical diseases including paroxysmal nocturnal hemoglobinuria and dysferlin-deficient muscular dystrophy [25], [26]. In this study, the soluble factors including factor H are provided by the normal human serum rather than from the KSHV-infected cells. Thus, the most likely factors that might mediate complement activation and are also regulated by KSHV in the infected cells are the membrane-associated factors. Furthermore, our immunohistochemistry results show that complement deposition is on the spindle tumor cells but not the adjacent uninvolved tissues, indicating that soluble factors are unlikely to be regulated by KSHV and be involved in the activation of the complement system. Indeed, we have found that complement regulatory proteins CD55 and CD59 are significantly downregulated in latently KSHV-infected endothelial cells and in KS spindle tumor cells. Importantly, we have shown that overexpression of either CD55 or CD59 in latently KSHV-infected cells is sufficient to abolish complement activation. These results are consistent with the facts that CD55 binds to C3b while CD59 binds to C5b-8 complex [21]. We have therefore concluded that KSHV activates the complement system by downregulating complement regulatory proteins during latent infection.

Following the initial exposure to normal human sera, most latently KSHV-infected cells are resistant to complement-mediated cytolysis indicating that the complement complexes (C5b-9) might be at sub-lytic dose or have not achieved full cytolytic function on these cells. We have detected deposition of factor H on the KSHV-infected cells, which are in agreement with the observation of C3b deposition on these cells. Factor H deposition cleaves C3b to generate C3d resulting in the formation of multimeric factor H-C3d complexes on the cell surface [45]. While we do not fully understand why the C5b-9 complexes are still formed, factor H deposition on these cells is likely important to provide protection to the cells because depletion of factor H sensitizes KSHV-infected cells to complement attack while reconstitution of the factor H-depleted serum with recombinant factor H rescue the KSHV-infected cells from complement attack. On the other hand, depletion of factor I has no effect on the sensitivity of KSHV-infected cells to complement attack, which is consistent with the lack of deposition of factor I on the KSHV-infected cells. Nevertheless, it is unclear how deposition of factor H might protect latently KSHV-infected cells from complement attack.

Activation of the complement system is implicated in chronic inflammation, cell lysis, and wound healing [21], [59], [60]. C5b-9 deposition has been detected in tissues of several chronic inflammatory diseases including systemic lupus erythematosus (SLE), diabetes, atherosclerosis, inflammatory bowel disease [17], [18], [20], [21]. C5b-9 deposition is also present in several types of cancer [21], [61]–[63]. Inflammatory cells can induce genomic instability, promote angiogenesis, and establish a favorable tumor microenvironment to facilitate tumor growth and survival through the production of pro-inflammatory and pro-angiogenic cytokines [64], [65]. Inflammatory cells can also promote neoplastic spread and metastasis of tumor cells by producing matrix metalloproteinases and chemokines [64], [65]. Because KS tumor is highly angiogenic containing vast inflammatory infiltration of immune cells [5], [66], it is likely that activation of the complement system could contribute to the pathological features of KS and the development of KS tumors.

We have shown that activation of the complement system activates the STAT3 pathway, which promotes the survival of latently KSHV-infected cells cultured in growth factor-depleted medium. Importantly, KSHV downregulation of CD55 and CD59 is required for C5b-9 deposition, STAT3 activation and the enhanced cell survival. KS tumors harbor high levels of pro-inflammatory and pro-angiogenic cytokines, some of which including IL-6 and oncostatin M, can induce STAT3 activation [67], [68]. It has also been shown that C5b-9 deposition on endothelial cells can release basic fibroblast growth factor and platelet-derived growth factor [69], both of which can activate STAT3 [70], [71]. Both basic fibroblast growth factor and platelet-derived growth factor are abundant in KS tumors and have been implicated in the pathogenesis of KS [72]–[75]. However, our results have shown that STAT3 tyrosine phosphorylation is involved with JAKs, which primarily mediates the activation of cytokine receptors, but not with Src, which primarily mediates the activation of growth factor receptors. Whether activation of the complement system induces pro-inflammatory and pro-angiogenic cytokines leading to STAT3 activation and enhanced cell survival remain to be further investigated [5].

A previous study has shown acute induction of STAT3 phosphorylation in endothelial cells within 30 min of contact with the C5b-9 complexes [76]. We did not observe increased STAT3 tyrosine phosphorylation until 16 h after the treatment with normal human serum. Thus, the mechanism of STAT3 activation described in our study is likely distinct from that of the previous study.

Acute KSHV infection induces STAT3 tyrosine phosphorylation [16] but its effect on long-term latently KSHV-infected cells is unclear. We have shown that long-term latent TIME-KSHV cells also have higher levels of STAT3 serine phosphorylation than TIME cells when cultured in full endothelial cell medium containing growth factors. When cultured in growth factor-depleted medium, only weak signal of tyrosine phosphorylation was detected in both TIME and TIME-KSHV cells. While weak constitutive serine phosphorylation was detected in TIME-KSHV but not TIME cells, which could be induced by kaposin B [48], it does not confer survival advantage for TIME-KSHV cells. In contrast, our results have shown that complement activation is required for a higher level of STAT3 tyrosine phosphorylation and for the enhanced cell survival in growth factor-depleted condition.

In summary, we have identified a distinct mechanism by which KSHV subverts the alternative complement pathway by downregulating complement regulatory proteins, which results in the activation of the STAT3 pathway and enhanced cell survival. The deregulation of the complement system might promote KSHV latency and contribute to persistent infection and development of KS tumors. Future studies should investigate the effect of the activated complement system on inflammation and angiogenesis in KS tumors.

## Materials and Methods

### Cell cultures and reagents

Telomerase-immortalized human umbilical vein endothelial cells (TIVE) and long-term KSHV-infected TIVE cells (TIVE-LTC) were obtained from Dr. Rolf Renne [39]. Telomerase-immortalized human microvascular endothelial cells (TIME) were obtained from Dr. Don Ganem [38]. KSHV BAC36-infected TIME (TIME-KSHV) cells were obtained by infection of TIME cells with BAC36 [37]. The infected cells were selected with hygromycin to achieve 100% stable latent infection based on the expression of LANA and other viral lytic proteins (Figure S1). This process usually took several weeks. TIME-KSHV cells were used after they had fully established latency but within less than 10 weeks following the initial establishment of latency. The cells were grown in medium without hygromycin for at least 1 week before the experiments. TIVE, TIVE-LTC, TIME and TIME-KSHV were grown in VascuLife VEGF Cell Culture Medium containing 2% FBS, L-glutamine, and growth factors including rhEGF, rhFGF basic, rhIGF-1, ascorbic acid, hydrocortisone, and heparin sulfate (Lifeline Cell Technology, Frederick, MD). KS lesions and other tissues were previously described [77]. Pooled complement human serum was purchased from Innovative Research, Inc (Novi, MI). C1q-depleted human serum, C3-depleted human serum, C6-depleted human serum, factor B-depleted human serum, purified C3 protein, and purified C6 protein were purchased from Quidel Corporation (San Diego, CA). Factor H-depleted human serum, factor I-depleted human serum, purified factor H protein were obtained from CompTech (Tyler, TX). Stattic (STAT3 inhibitor), JAK inhibitor I, LY294002 (PI3K inhibitor), SB203580 (p38 inhibitor), U0126 (MEK inhibitor), U-73122 (Phospholipase C inhibitor), and Src inhibitor I were purchased from Calbiochem (San Diego, CA).

### Immunohistochemistry

Formalin-fixed 3- or 4-µm serial sections from paraffin-embedded, formalin-fixed human KS lesions were deparaffinized in xylene, washed with 100% ethanol, and then rehydrated in 95% ethanol. Hydrogen peroxidase (3%) in absolute methanol was used to quench endogenous peroxidase. Antigen retrieval was performed by microwaving the tissue sections in citrate buffer (pH 6.0) for 30 min followed by cooling at room temperature for 20 min. After blocking with normal serum, the slides were incubated with primary antibodies. A rat monoclonal antibody to LANA (LNA-1) used at 1∶100 dilution was purchased from Advanced Biotechnologies Inc (Columbia, MD). A rabbit polyclonal antibody to CD55 (sc-9156) was obtained from Santa Cruz (Santa Cruz, CA). A mouse monoclonal antibody to CD59 (MEM-43/5) and rabbit polyclonal antibodies to C5b-9 (ab55811) and C3d (ab15981) were from Abcam (Cambridge, MA). All primary antibodies were used at 1∶100 dilution. After washing with PBS, slides were incubated with peroxidase-conjugated secondary antibodies, and signals were revealed with 0.03% diaminobenzidine as chromogen and counterstained with hematoxylin.

### Immunofluorescence assay (IFA)

Cell monolayers were fixed in 4% paraformaldehyde for 10 min and washed with PBS. The fixed cells were permeabilized with 0.25% Triton X-100 in PBS for 15 min. After blocking in PBS containing 3% BSA for 30 min at 4°C, samples were incubated overnight at 4°C with primary antibody at 1∶200 in PBS containing 3% BSA. After washing with PBS containing 0.05% Tween (PBS-T), samples were incubated for 15 min with AlexaFluor conjugated secondary antibodies at 1∶500 in PBS containing 3% BSA, washed again and incubated with 0.5 µg/ml 4-,6-diamidino-2-phenylindole (DAPI) in PBS for 1 min. Samples were mounted in FluorSave Reagent (Calbiochem, San Diego, CA). Pictures were obtained using an IX71 Olympus Fluorescence Microscope equipped with a digital camera or a Nikon ECLIPSE Ti Confocal Laser-Scanning Microscope. Nikon NIS Elements Ar Microscope Imaging Software was used for image analysis and 3-D rendering.

In addition to antibodies to LANA, C5b-9, CD55 and CD59, mouse monoclonal antibodies to C3b (755, Abcam), K8.1 (13-213-100, Advanced Biotechnologies, Inc), ORF59 (13-211-100, Advanced Biotechnologies, Inc), factor H (OX-24, Abcam), factor I (OX-21, Abcam), and integrin αVβ3 (LM609, Millipore, Billerica, MA) were also used as primary antibodies. A mouse monoclonal antibody to CD55 (MEM-118, Abcam) was used for dual staining with C5b-9. A mouse monoclonal antibody to ORF65 was previously described [37]. Secondary antibodies included AlexaFluor 568 anti-rabbit IgG, AlexaFluor 568 anti-rat IgG, AlexaFluor 568 anti-mouse IgG, AlexaFluor 647 anti-mouse IgG, and AlexaFluor 647 anti-mouse IgG1, all from Invitrogen (Carlsbad, CA).

### Activation of the complement system and quantification of C5b-9 and C3b depositions

Cells at 50,000 cells per well were seeded in 24-well plates with 12-mm glass coverslip overnight. Human complement serum were mixed with VascuLife VEGF Cell Culture Medium and the mixture was applied to the cells. Heat-inactivation was performed at 56°C for 30 min. For reconstitution of complement factor-depleted human serum, the complement-depleted human serum was mixed with the purified complement factor protein. Based on the certificate of analysis of the serum from the manufacturer, appropriate amounts of the purified complement factor was added to the complement factor-depleted human serum to achieve the functional activity ratio of CH50/ml that is equivalent to normal human serum. To identify the complement pathway, complement activation was also performed in the presence of 10 mM EGTA and 20 mM MgCl_2_ or 20 mM EDTA. After treatment of human complement serum, the cover slip was washed once with PBS then stained by immunofluorescence assay to detect C5b-9 deposition. Images were quantified for C5b-9 deposition using the particle analyzing function of the ImageJ software (National Institute of Health, Bethesda, MD). To detect C3b deposition, cells were grown in 6-well plates. Following complement treatment, cells were detached and quantified by flow cytometry analysis.

### Quantitative real-time reverse transcription PCR (RT-qPCR)

Total RNA from cultured cells was isolated with NucleoSpin RNA II as recommended by the manufacturer (MACHEREY-NAGEL Inc., Bethlehem, PA). Total RNA of 1 µg was reverse-transcribed in a total volume of 20 µl to obtain first-strand cDNA using the Superscript III first-strand synthesis system (Invitrogen), according to the manufacturer's instructions. Real-time PCR was performed using Power SYBR Green PCR Master Mix (Applied Biosystems, Foster City, CA) and a RealPlex thermal cycler (Eppendorf, New York, NK). The following PCR program was used: 95°C for 10 min, 40 cycles of 95°C for 15 s, 60°C for 1 min. All samples, including a control without the template were examined in triplicate for each primer pair. Amplification for human glyceraldehyde 3-phosphate dehydrogenase (GAPDH) was used as a loading control. Data analysis was performed as previously described [78]. The primers used in this study were hGAPDHs: 5′-TGACAACAGCCTCAAGAT-3′ and hGAPDHas: 5′-GAGTCCTTCCACGATACC-3′ for GAPDH; hCD59s: 5′-AAGAAGGACCTGTGTAACTT-3′ and hCD59as: 5′ GAGTCACCAGCAGAAGAA-3′ for CD59; hCD55s: 5′-GGCAGTCAATGGTCAGATA-3′ and hCD55as: 5′-GGCACTCATATTCCACAAC-3′ for CD55; and hCD46s: 5′ TTTGAATGCGATAAGGGTTT-3′ and hCD46as: 5′ GAGACTGGAGGCTTGTAA-3′ for CD46. The primers were synthesized by Integrated DNA Technologies (Coralville, IA).

### Western blot analysis

Cells were lysed in RIPA buffer containing 10 mM Tris-HCl at pH 8.0, 1 mM EDTA, 140 mM NaCl, 0.1% SDS, 0.1% sodium deoxycholate, 1% Triton X-100 and a protease inhibitor cocktail (AMRESCO LLC, Solon, OH). The cell suspension was incubated at 4°C for 15 min and then centrifuged at 14,000 g for 15 min at 4°C. The supernatant was collected and the protein concentration was measured by BCA assay (Pierce, Rockford, IL). The protein was separated by electrophoresis on SDS-PAGE and transferred onto a nitrocellulose membrane (GE Healthcare, Piscataway, NJ). Immunodetection was performed with the mouse monoclonal antibodies to CD46 (MEM-258, Abcam) and β-tubulin (7B9, Sigma, St. Louis, MO), rabbit polyclonal antibodies to STAT3 (#9132, Cell Signaling Technology, Danvers, MA) and phospho-STAT3 Ser727 (9134, Cell Signaling Technology), and a rabbit monoclonal antibody to phopho-STAT3 Tyr705 (D3A7, Cell Signaling Technology) in addition to the antibodies to CD55 and CD59, all used at 1∶1000 dilution.

### Flow cytometry

Cells detached from the plate with 5 mM EDTA in Dulbecco's phosphate-buffered saline were fixed with 4% paraformaldehyde in PBS for 20 min and incubated with primary antibodies at 1∶50 dilution (CD55) or 1∶500 (C3b and C59) for 30 min at 4°C. For the detection of C3b, complement activation was carried as described above before cell detachment. After washing in PBS, cells were incubated with APC-conjugated goat anti-mouse or rabbit antibodies. Flow cytometry was performed with a FACS Canto II flow cytometer and analyzed with CellQuest Pro software (Becton Dickinson, Bedford, MA). Prior to analysis, all samples were gated to eliminate dead cells. Besides antibodies to CD55 and CD59, a mouse monoclonal antibody to C3b (10C7, Abcam) was used for the analysis.

### Cell viability assay

After washing the cells with Dulbecco's phosphate-buffered saline, 5 µM EthD-1 (Invitrogen) in EGM was applied. After incubation for 30 min at 37°C, the total numbers of live cells and the labeled dead cells were observed and counted under an IX71 Olympus Fluorescence Microscope.

### MTT assay

Cells at 5,000 cells/well were seeded in 96-well plates overnight. Following treatment with human serum complement with and without the presence of chemical inhibitors, cells were incubated in 0.5 mg/ml of MTT solution (Sigma) at 37°C for 4 h. The solution was then replaced with DMSO. The optical densities were determined at 540 nm and 630 nm using a Synergy TM 2 Multi-Mode Microplate Reader (BioTek, Winooski, VT).

### Lentiviruses and overexpression of CD55 and CD59

Lentiviral plasmids expressing CD55 (h-CD55 Lentivirus vector, #LV112276), CD59 (h-CD59 Lentivirus vector, #LV112288) and the blank vector (pLenti-GIII-CMV, #LV587) were purchased from Applied Biological Materials Inc (Richmond, BC). Infectious Lentiviruses were produced using ViraPower Lentiviral Packaging Mix (Invitrogen). Specifically, 293T cells were seeded at 8×10^6^ cells in a 150 cm^2^ flask overnight. DNAs from 6 µg of pLP1, 6 µg of pLP2, 6 µg of pLP/VSVG and 9 µg of the target gene plasmid were transfected into cells using Lipofectamine 2000 Transfection Reagent according to the instructions of the manufacturer (Invitrogen). The supernatant containing Lentivirus was harvested 48 h later, filtered through a 0.45-µm-pore-size filter, and concentrated by centrifugation at 24,000 rpm in a Beckman SW32 rotor for 2 h at 4°C. The pellet containing the virus was resuspended in Opti-MEM reduced serum medium (Invitrogen). Viruses were then stored at −80°C for long-term use or 4°C for short-term use.

To generate stable cells expressing CD55 or CD59, TIME-KSHV cells were infected with ∼10 MOI of the Lentivirus in the presence of 8 µg/ml of polybrene. Two days after infection, the infected cells were selected with 1 µg/ml of puromycin for 2 weeks. The cells were then cryo-frozen or used for other experiments.

### Statistics

Results are shown as mean ± SD (standard deviations) where appropriate. The 1-tailed Student's test was used to compare data between the different groups. Statistical significance assumed at P values less than 0.05, 0.01 or 0.001 is represented by “*”, “**” or “***” respectively.

## Supporting Information

Figure S1 **TIME-KSHV cells are latently infected by KSHV.** Immunofluorescence staining of KSHV latent protein LANA, and lytic proteins K8.1 and ORF65 in TIME and TIME-KSHV cells. BCBL-1 cells were used as controls. The scale bar is 100 µm.
(TIF)

Figure S2 **Randomly selected images of C5b-9 deposition from TIME and TIME-KSHV cells.** Cells were incubated with 10% normal human serum for 30 min and stained for C5b-9 by immunofluorescence assay. TIME-KSHV cells harbor BAC36 containing a GFP cassette. CD59 staining was done on TIME cells and captured in the far-red channel but pseudo-colored in green to show the cell shape and facilitate the localization of C5b-9 deposition. These images were quantified and the average C59-b positive areas per cell were shown in Figure 2B. Scale bar is 100 µm.
(EPS)

Figure S3 **Cell surface localization of C5b-9 deposition on TIME-KSHV cells.** TIME and TIME-KSHV cells were incubated with 10% normal or heat-inactivated human serum for 30 min, and stained for C5b-9 (red), integrin αVβ3 (green) to label plasma membrane and DAPI (blue) to localize the nucleus. Z-stack images were acquired with confocal laser-scanning microscopy. Three-dimensional software was used to generate z-projection images from at least 70 confocal images of 0.1 µm sections. The 3-D images (XY panels) were rotated on the x-axis (XZ panels) and y-axis (YZ panels) to visualize C5b-9 localization on the cell membrane. Arrows show representative areas of C5b-9 depositions on cell surfaces.
(EPS)

Figure S4 **Complement activation in latently KSHV-infected cells does not induce the expression of KSHV lytic proteins.** (A–B) TIME-KSHV cells were incubated with 10% normal human serum for 1 h and then stained for C5b-9 deposition (red) and KSHV lytic proteins (magenta) ORF59 (A) or ORF65 (B). BCBL-1 cells were used as positive controls. The scale bar is 20 µm.
(EPS)

Figure S5 **Detection of C5b-9 deposition on latently KSHV-infected TIVE-LTC but not uninfected TIVE cells.** Cells incubated with 10% normal human serum for 30 min were stained for C5b-9 deposition (red) and actin (green) by immunofluorescence assay. The scale bar is 20 µm.
(EPS)

Figure S6 **Factor B but not C1q was required for C5b-9 deposition on TIME-KSHV cells.** TIME or TIME-KSHV cells were incubated with factor B- or C1q-depleted human serum for 30 min. The C5b-9 deposition was detected by immunofluorescence staining. The scale bar is 100 µm.
(EPS)

Figure S7 **KSHV ORF4 is not expressed in TIME-KSHV cells before and after complement activation.** KSHV ORF4 mRNA was examined by RT-PCR in TIME-KSHV cells with and without treated with heat-inactivated or normal human serum for 1 h. TIME cells and TPA-induced BCBL-1 cells were used as negative and positive controls, respectively. ORF72 mRNA was also examined to show the expression of the viral latent gene.
(EPS)

Figure S8 **No change of CD55 and CD59 expression in latently KSHV-infected cells following complement activation.** (A and B) CD55 and CD59 expression examined by immunofluorescence staining in TIME-KSHV cells remained low following complement activation. Cells were incubated with normal human serum for 1 h and then co-stained for C5b-9 deposition (red) and CD55 or CD59 (magenta). (C and D) Complement activation did not affect the expression of CD55 and CD59 proteins. TIME-KSHV cells were either untreated or incubated with normal human serum for 1 h and then examined for the total protein levels of CD55 (C) or CD59 (D) by Western-blotting. The scale bar is 20 µm.
(EPS)

Figure S9 **No correlation between the amount of C5b-9 deposition and cell killing in TIME-KSHV cells following complement activation.** TIME-KSHV cells were incubated with 10% normal human serum for 1 h and co-stained for C5b-9 deposition and EthD-1 to identify dead cells. Twenty cells were randomly selected from both live and dead cells, and quantified for the average C5b-9 positive areas per cell. The results were shown in Figure S10.
(TIF)

Figure S10 **No correlation between the amount of C5b-9 deposition and cell killing in TIME-KSHV cells following complement activation.** The images in Figure S9 were quantified for C5b-9 deposition using the ImageJ software. Each dot represents the analyzed value from one individual cell and the average value of 20 analyses was indicated as a horizontal black bar. No significant difference was found by Student's *t*-test.
(EPS)

Figure S11 **The enhanced cell survival of latently KSHV-infected endothelial cells by complement is mediated by the STAT3 pathway.** TIME-KSHV cells were cultured for 48 h in normal human serum in growth factor-depleted medium with and without JAK or STAT3 inhibitor, and stained with EthD-1 to identify the dead cells. Cells cultured in heat-inactivated human serum were used as controls. The scale bar is 200 µm.
(TIF)

Figure S12 **The enhanced cell survival of TIME-KSHV cells by complement activation is not mediated by PI3K, p38, ERK, PLC and Src pathways.** TIME-KSHV cells were cultured for 48 h in normal human serum in growth factor-depleted medium with and without the indicated inhibitors, and stained with EthD-1 to identify the dead cells. Cells cultured in 10% heat-inactivated human serum were used as controls. The scale bar is 200 µm.
(TIF)

Figure S13 **STAT3 inhibitor does not affect C5b-9 deposition on TIME-KSHV cells.** (A) The effect of STAT3 inhibitor on C5b-9 deposition. TIME-KSHV cells were untreated or treated with STAT3 inhibitor for 16 h, incubated with normal human serum for 30 min, and stained for C5b-9 deposition. The scale bar is 50 µm. (B) Quantification of C5b-9 deposition. Three images from each treatment shown in (A) were analyzed for C5b-9-positive areas using the ImageJ software. The average C5b-9 staining area per cell was calculated by dividing the total pixels of C5b-9 staining in a microscopic field by the cell number. The results were shown in means and error bars. No significant difference was found by Student's *t*-test.
(EPS)

Figure S14 **C5b-9 deposition was abolished on TIME-KSHV cells following overexpression of either CD55 or CD59.** TIME-KSHV cells with stable overexpression of CD55, CD59 or vector control were incubated with 10% normal human serum for 1 h and stained for C5b-9 by immunofluorescence staining. TIME and TIME-KSHV cells were used as negative and positive controls, respectively. Ten images were randomly selected from each cell type. These images were quantified and the average C59-b positive areas per cell were shown in Figure 8D.
(EPS)
