# Supplementary figures and images for: Exploitation of the Complement System by Oncogenic Kaposi's Sarcoma-Associated Herpesvirus for Cell Survival and Persistent Infection
Source: PLoS Pathog. 2014 Sep 25;10(9):e1004412. doi: 10.1371/journal.ppat.1004412 (PMC4177982; doi:10.1371/journal.ppat.1004412)

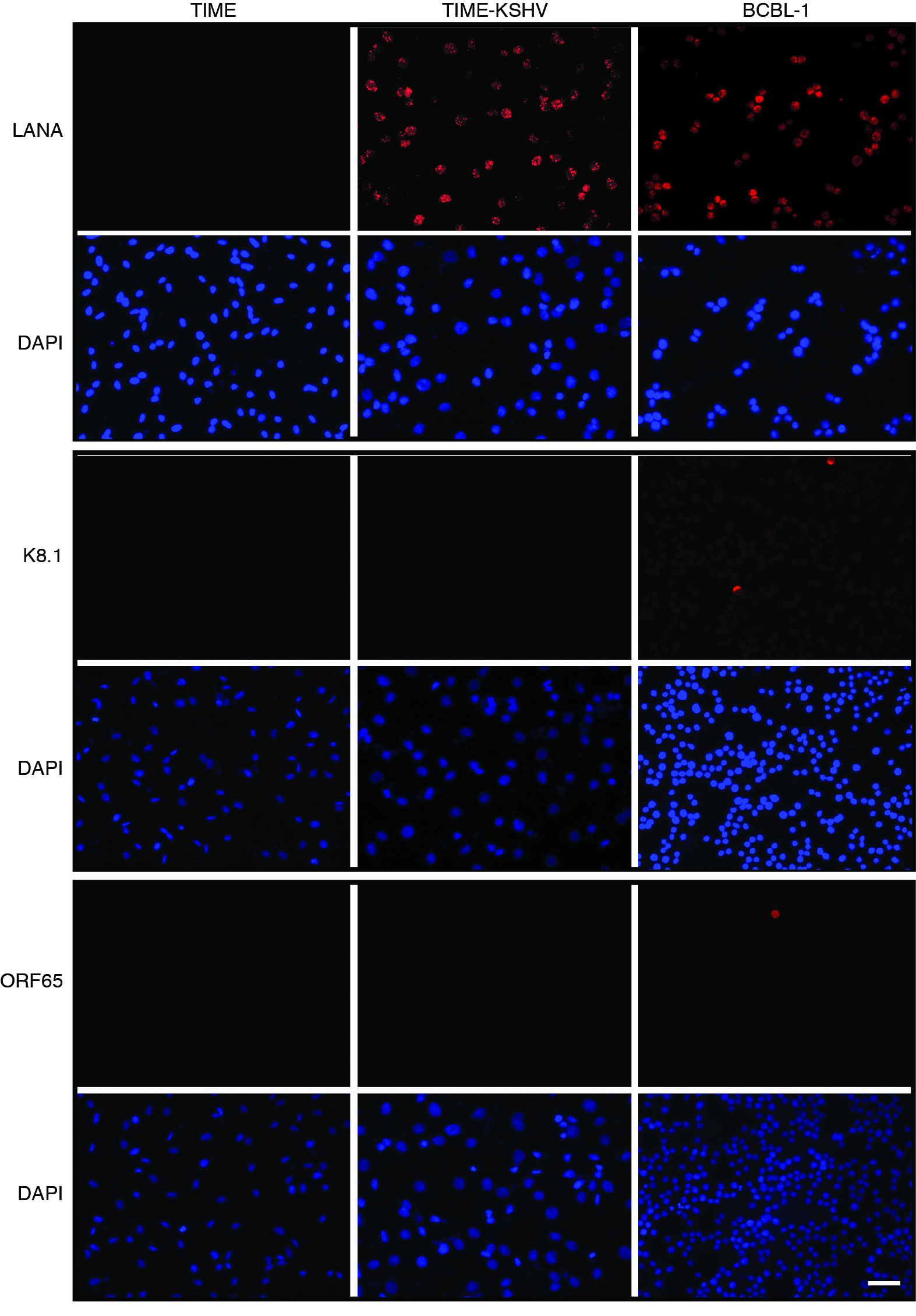

Supplement: Figure S1 — TIME-KSHV cells are latently infected by KSHV. Immunofluorescence staining of KSHV latent protein LANA, and lytic proteins K8.1 and ORF65 in TIME and TIME-KSHV cells. BCBL-1 cells were used as controls. The scale bar is 100 µm. (TIF) [file ppat.1004412.s001.tif]

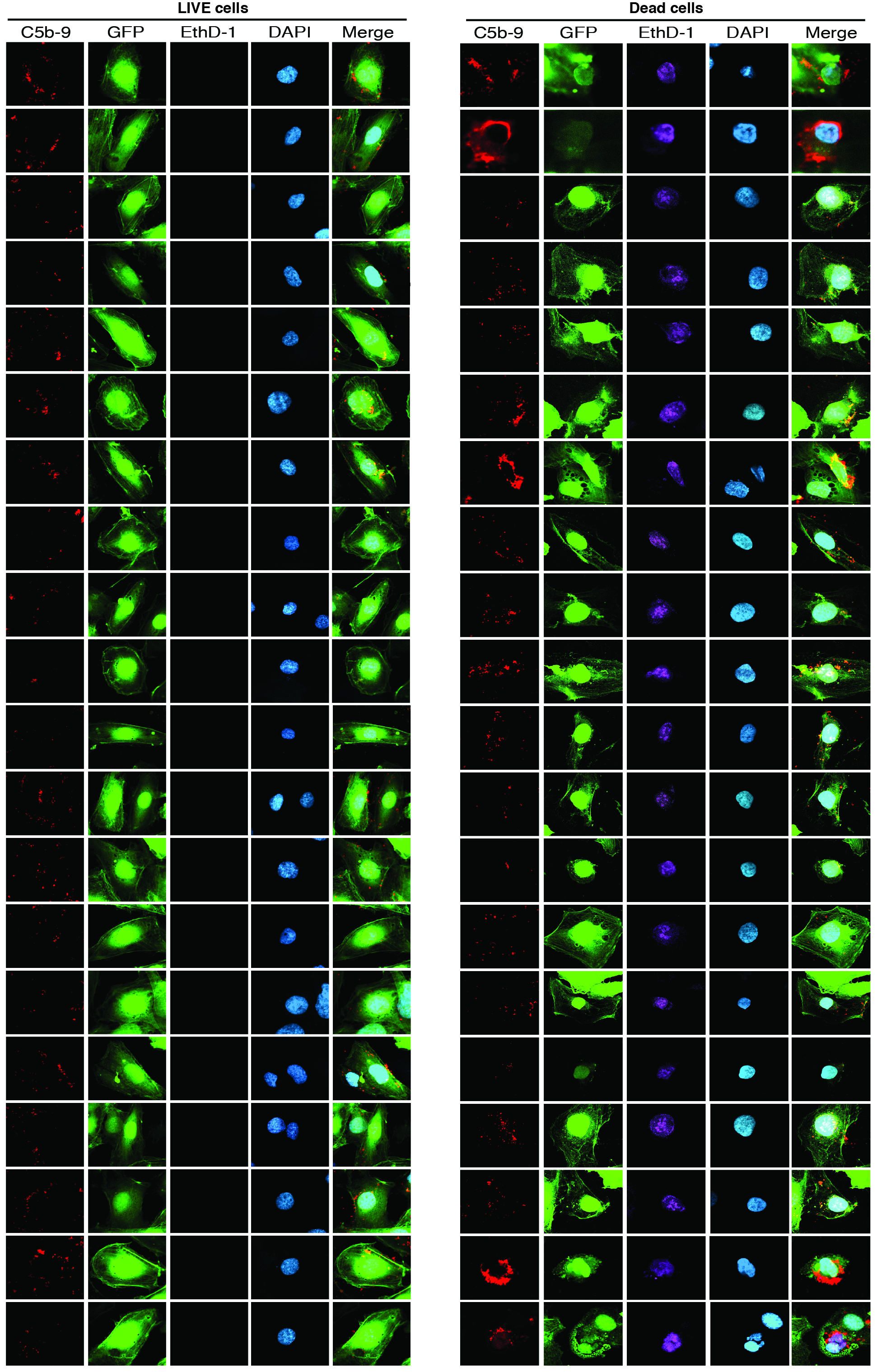

Supplement: Figure S9 — No correlation between the amount of C5b-9 deposition and cell killing in TIME-KSHV cells following complement activation. TIME-KSHV cells were incubated with 10% normal human serum for 1 h and co-stained for C5b-9 deposition and EthD-1 to identify dead cells. Twenty cells were randomly selected from both live and dead cells, and quantified for the average C5b-9 positive areas per cell. The results were shown in Figure S10. (TIF) [file ppat.1004412.s009.tif]

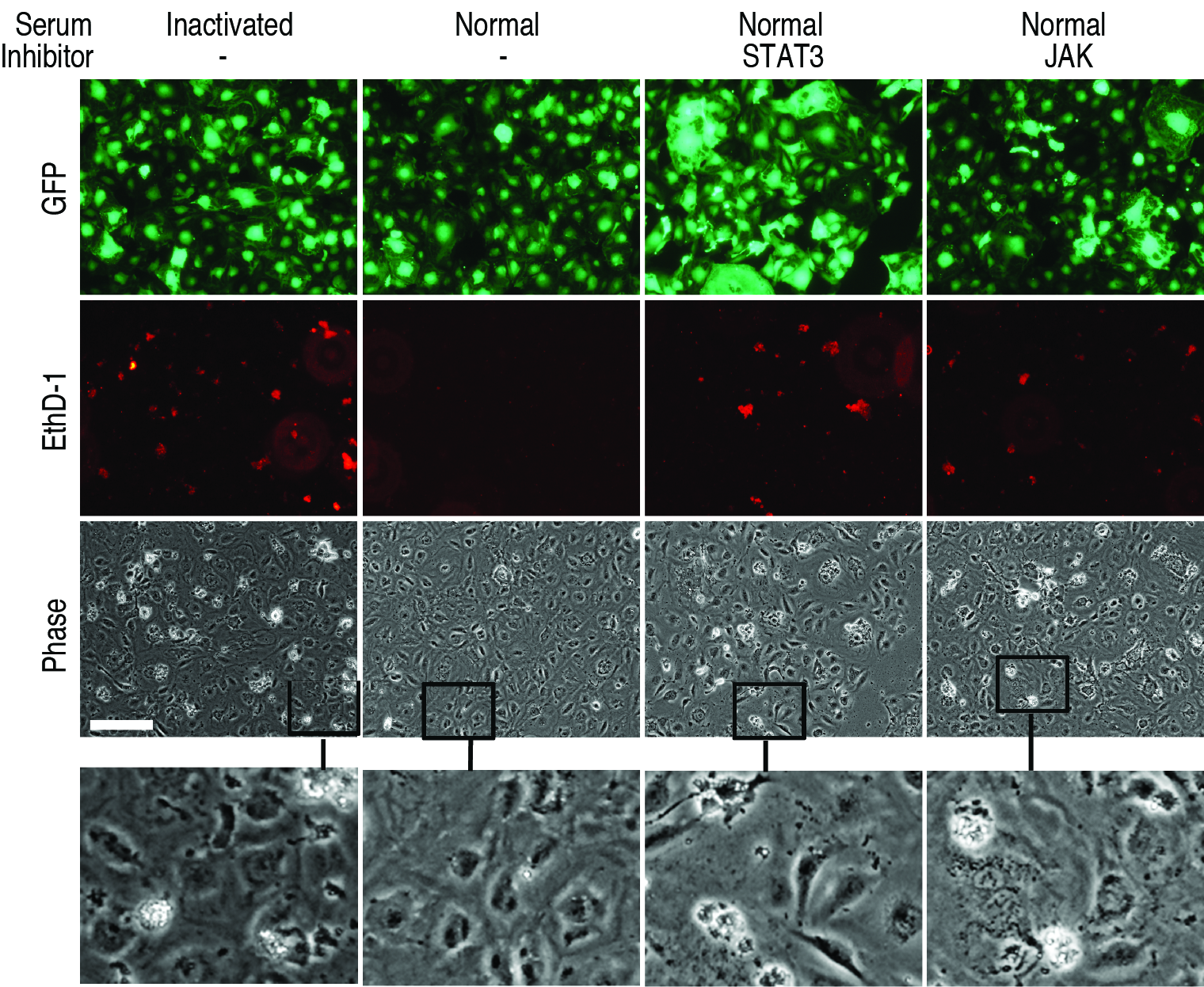

Supplement: Figure S11 — The enhanced cell survival of latently KSHV-infected endothelial cells by complement is mediated by the STAT3 pathway. TIME-KSHV cells were cultured for 48 h in normal human serum in growth factor-depleted medium with and without JAK or STAT3 inhibitor, and stained with EthD-1 to identify the dead cells. Cells cultured in heat-inactivated human serum were used as controls. The scale bar is 200 µm. (TIF) [file ppat.1004412.s011.tif]

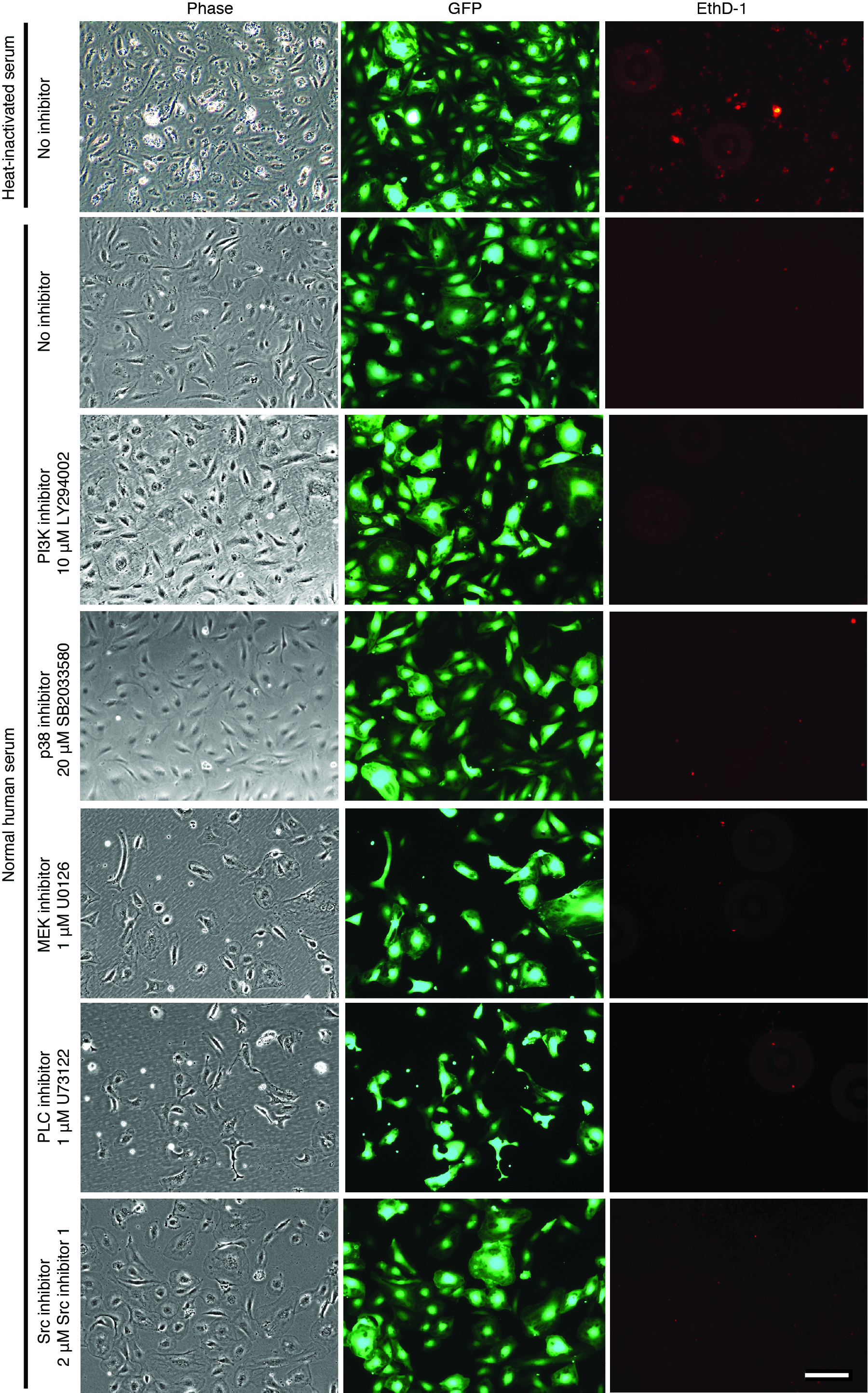

Supplement: Figure S12 — The enhanced cell survival of TIME-KSHV cells by complement activation is not mediated by PI3K, p38, ERK, PLC and Src pathways. TIME-KSHV cells were cultured for 48 h in normal human serum in growth factor-depleted medium with and without the indicated inhibitors, and stained with EthD-1 to identify the dead cells. Cells cultured in 10% heat-inactivated human serum were used as controls. The scale bar is 200 µm. (TIF) [file ppat.1004412.s012.tif]
